# Effects of saline or albumin fluid bolus in resuscitation: evidence from re-analysis of the FEAST trial

**DOI:** 10.1016/S2213-2600(19)30114-6

**Published:** 2019-07

**Authors:** Michael Levin, Aubrey J Cunnington, Clare Wilson, Simon Nadel, Hans Joerg Lang, Nelly Ninis, Mignon McCulloch, Andrew Argent, Heloise Buys, Christopher A Moxon, Abigail Best, Ruud G Nijman, Clive J Hoggart

**Affiliations:** a Section of Paediatrics, Division of Infectious Diseases, Department of Medicine, Imperial College London, London, UK; b Department of Paediatric Intensive Care, St Mary's Hospital, Imperial College Healthcare NHS Trust, London, UK; c Paediatric Referent, Médecins sans Frontières, Brussels, Belgium; d Department of Paediatrics, St Mary's Hospital, Imperial College Healthcare NHS Trust, London, UK; e Department of Paediatric Emergency Medicine, St Mary's Hospital, Imperial College Healthcare NHS Trust, London, UK; f Red Cross Children's Hospital, Cape Town, South Africa; g Wellcome Centre for Integrative Parasitology, Institute of Infection, Immunity and Inflammation, University of Glasgow, Glasgow, UK; h Malawi–Liverpool–Wellcome Trust Clinical Research Programme, College of Medicine, University of Malawi, Blantyre, Malawi; i Institute of Infection and Global Health, University of Liverpool, Liverpool, UK

## Abstract

**Background:**

Fluid resuscitation is the recommended management of shock, but increased mortality in febrile African children in the FEAST trial. We hypothesised that fluid bolus-induced deaths in FEAST would be associated with detectable changes in cardiovascular, neurological, or respiratory function, oxygen carrying capacity, and blood biochemistry.

**Methods:**

We developed composite scores for respiratory, cardiovascular, and neurological function using vital sign data from the FEAST trial, and used them to compare participants from FEAST with those from four other cohorts and to identify differences between the bolus (n=2097) and no bolus (n=1044) groups of FEAST. We calculated the odds of adverse outcome for each ten-unit increase in baseline score using logistic regression for each cohort. Within FEAST participants, we also compared haemoglobin and plasma biochemistry between bolus and non-bolus patients, assessed the effects of these factors along with the vital sign scores on the contribution of bolus to mortality using Cox proportional hazard models, and used Bayesian clustering to identify subgroups that differed in response to bolus. The FEAST trial is registered with ISRCTN, number ISRCTN69856593.

**Findings:**

Increasing respiratory (odds ratio 1·09, 95% CI 1·07–1·11), neurological (1·26, 1·21–1·31), and cardiovascular scores (1·09, 1·05–1·14) were associated with death in FEAST (all p<0·0001), and with adverse outcomes for specific scores in the four other cohorts. In FEAST, fluid bolus increased respiratory and neurological scores and decreased cardiovascular score at 1 h after commencement of the infusion. Fluid bolus recipients had mean 0·33 g/dL (95% CI 0·20–0·46) reduction in haemoglobin concentration after 8 h (p<0·0001), and at 24 h had a decrease of 1·41 mEq/L (95% CI 0·76–2·06; p=0·0002) in mean base excess and increase of 1·65 mmol/L (0·47–2·8; p=0·0070) in mean chloride, and a decrease of 0·96 mmol/L (0·45 to 1·47; p=0·0003) in bicarbonate. There were similar effects of fluid bolus in three patient subgroups, identified on the basis of their baseline characteristics. Hyperchloraemic acidosis and respiratory and neurological dysfunction induced by saline or albumin bolus explained the excess mortality due to bolus in Cox survival models.

**Interpretation:**

In the resuscitation of febrile children, albumin and saline boluses can cause respiratory and neurological dysfunction, hyperchloraemic acidosis, and reduction in haemoglobin concentration. The findings support the notion that fluid resuscitation with unbuffered electrolyte solutions may cause harm and their use should be cautioned. The effects of lower volumes of buffered solutions should be evaluated further.

**Funding:**

Medical Research Council, Department for International Development, National Institute for Health Research, Imperial College Biomedical Research Centre.

## Introduction

Both adult and paediatric resuscitation guidelines recommend boluses of intravenous fluid, most commonly crystalloid or colloid solutions, with 20–60 mL/kg bodyweight being recommended in the first hour of resuscitation.^1^, ^2^ The practice of volume expansion by rapid bolus infusion was introduced not on the basis of evidence from randomised trials, but on current understanding of the physiology of shock. Patients with sepsis or other forms of shock, whether arising from external blood or fluid losses, internal fluid loss from capillary leakage, or pathological vasodilatation, are thought to be hypovolaemic and thus to have diminished venous return to the heart. Clinical experience has supported this physiological concept because bolus resuscitation is usually associated with improved pulse pressure, warming of the peripheries, and increased urine output. However, clinicians are also aware that some patients deteriorate, rather than improve, after volume resuscitation. Furthermore, patients receiving large volumes of fluid are known to develop generalised oedema,^1^, ^2^ often accompanied by increased requirement for high ventilation pressures, unless fluid is removed by haemofiltration or diuresis. Despite these known adverse effects of fluid, bolus fluid resuscitation is so widely accepted in the practice of emergency medicine in resource-rich countries that neither clinicians nor research ethics committees are likely to agree to test the practice in a randomised controlled trial with a no-fluid bolus control.

**Research in context**

**Evidence before this study**

Fluid resuscitation has been recommended initial treatment of sepsis and septic shock for more than 40 years. The practice of fluid resuscitation in sepsis has strong theoretical and physiological justification, because volume expansion is believed to correct the hypovolaemia occurring in sepsis due to capillary leak and pathological vasodilatation, and thus increases cardiac output by achieving a more favourable position on the Starling curve.

We searched PubMed from Sept 1, 1974, to July 31, 2018, using the search term “fluid resuscitation in sepsis” restricting the search to human studies, and English language.

We identified 1827 publications, including reviews, observational studies, and clinical trials. We found numerous observational studies and randomised trials comparing different types of fluid resuscitation, including colloids (albumin, gelatin, dextrans, and starch) and different crystalloids (normal saline, hypertonic saline, Ringer's lactate, and other balanced salt solutions). We identified only one randomised controlled trial (the FEAST trial) in which volume resuscitation by itself was compared with maintenance fluids alone. In the FEAST trial, more than 3000 children with fever and clinical signs of impaired perfusion were randomly assigned to volume resuscitation with boluses of 5% albumin or normal saline, or maintenance fluids alone. The trial was stopped because of increased mortality in the bolus groups. Since FEAST is the only randomised controlled trial comparing bolus volume expansion with a no bolus control group, and showed harm from fluid expansion, it provides unique data through which to understand the physiological effects of fluid resuscitation.

**Added value of this study**

The findings of the FEAST trial—that volume expansion with 20-40 mL/kg of normal saline or albumin increased mortality in critically ill children, compared with no bolus—has been intensively debated by the intensive care and resuscitation community, and has left practitioners uncertain as to what the best resuscitation practice should be. To date, there has been no clear explanation as to why volume resuscitation was harmful in FEAST. Because FEAST was done in resource-poor hospitals in Africa, where other aspects of intensive care were not available, including mechanical ventilation and inotropic drugs, it provides unique data about the physiological effects of bolus volume expansion without the interference of other intensive care methods.

We compared sequential haemodynamic and vital sign data, haemoglobin, and blood chemistry in the bolus and no bolus groups of FEAST. Using novel composite scores to compare changes in respiratory, cardiovascular, and neurological function between the bolus and no bolus groups, we found that volume expansion with either 5% albumin or normal saline caused worsening of respiratory function and increased signs of raised intracranial pressure, but improved cardiovascular function. Bolus recipients had lower haemoglobin concentrations at 8 h, lower bicarbonate, and increased base deficit and chloride at 24 h after the bolus infusion. Regression modelling using the physiological variables significantly altered by bolus suggest that the increased mortality in FEAST can be explained by bolus-induced worsening in respiratory and neurological function, haemodilution, and hyperchloraemic acidosis.

**Implications of all the available evidence**

Despite the universal belief that volume expansion with crystalloid or colloidal fluid is beneficial in sepsis, and the inclusion of bolus volume expansion in current treatment recommendations, the FEAST trial showed fluid bolus increased mortality. The results of our analysis of the vital signs, haematological data, and biochemical data from FEAST provide a biologically plausible explanation for the adverse outcomes associated with bolus fluids. A modest bolus (20-40 mL/kg) of normal saline or albumin resulted in prolonged hyperchloraemic acidosis, reduced haemoglobin concentration, and worse respiratory and neurological function in the early hours after infusion, despite transient improvement in cardiovascular function, and explained the increase mortality in bolus recipients. Of these effects, saline-induced hyperchloraemic acidosis was a major contributor to the adverse outcome in bolus recipients. Our data suggest that normal saline and other unbuffered crystalloid solutions should be avoided in resuscitating seriously ill patients. Because volume resuscitation is associated with deterioration of respiratory function and neurological function in some patients, caution in use of fluids might be needed in patients with respiratory or CNS compromise, and trials of lower volumes of buffered solutions are now needed to establish whether the beneficial effects of fluid on cardiovascular function can be achieved with less risk of respiratory and neurological compromise.

By contrast with practice in resource-rich countries, fluid bolus resuscitation is not routine in Africa and other resource-poor regions. There has been an equipoise in many African hospitals between clinicians who recommend fluid resuscitation and those who avoid rapid fluid bolus resuscitation for fear that it might result in pulmonary or cerebral oedema. It was this equipoise in the use of fluids for resuscitation that motivated the FEAST (Fluid Expansion As Supportive Treatment) trial.^3^ In FEAST, 3141 febrile children with signs of under-perfusion in Uganda, Kenya, and Tanzania were randomly allocated to receive 20–40 mL/kg of normal saline or albumin bolus or maintenance only fluids. There was surprise both among the investigators as well as the wider scientific community when the trial was stopped by the study data and safety monitoring group after 3141 patients had been recruited, because of clear evidence of increased incidence of death in the recipients of bolus fluids.^3^

Since publication of the FEAST trial in 2011, there has been extensive debate about the generalisability of the findings and their implications for practice.^4^, ^5^, ^6^, ^7^, ^8^, ^9^, ^10^, ^11^, ^12^, ^13^, ^14^ The surprising finding, that a common medical intervention increased mortality in the only randomised controlled trial ever done to test fluid resuscitation against a no bolus control, has raised several important questions. What were the mechanisms by which fluid resuscitation increased mortality in FEAST? Were the findings specific to the population of patients in east Africa included in the study, or are they relevant to patients globally? Were there subgroups of patients included in the FEAST trial who were adversely affected by bolus, whereas others experienced benefit? Was the adverse effect due to the nature of the fluids used (normal saline and 5% albumin, both high chloride solutions) rather than the volume infused? Should international recommendations for fluid resuscitation be altered on the basis of this trial?

Research ethics committees in Africa and other resource-poor regions are unlikely to approve further trials of fluid resuscitation in light of the increased mortality shown in FEAST. Conversely, neither clinicians nor ethics committees in most resource-rich countries are likely to approve a trial in which fluid bolus is withheld from critically ill patients, because of the widely held belief in the benefits of fluid resuscitation. Many clinicians involved in emergency care in Europe and North America have chosen to disregard the results of the FEAST trial as being unique to the African context and argue that the findings are not relevant in well resourced settings, where the availability of ventilators and inotropes can ameliorate any negative effects of bolus fluids.^9^ The findings from FEAST have thus confused current clinical practice in resuscitation, raising the question of whether the current standard intervention to rapidly expand circulating volume is harmful and perhaps increases deaths and admissions to intensive care.

In light of the unresolved questions raised by FEAST, we believed that there was both a need and an obligation to fully use all the available data from the FEAST trial to understand why fluid bolus was associated with increased mortality. We postulated that the increased mortality in bolus recipients resulted from a measurable adverse effect of bolus fluids on cardiovascular function, respiratory function, raised intracranial pressure or neurological function, oxygen carrying capacity, or the biochemical and acid base status of bolus recipients. To test this hypothesis we developed composite scores to measure respiratory function, cardiovascular function, and identify raised intracranial pressure, using sequential vital sign data from FEAST, and compared the function of each system, as well as plasma biochemistry and acid-base balance, in the bolus and no bolus groups of FEAST.

## Methods

### Study design

**Figure 1:**
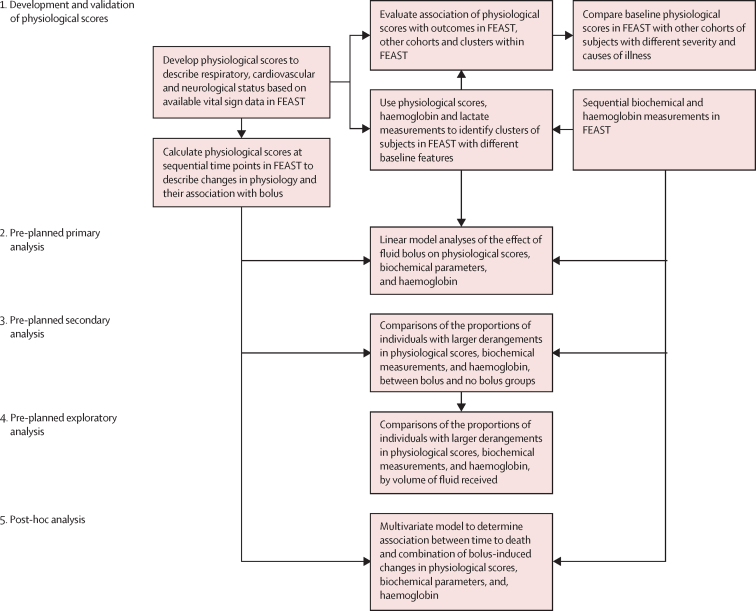
Study overview We developed physiological scores to describe respiratory, neurological, and cardiovascular function, and compared them between FEAST and four additional cohorts of ill children. We assessed their relationships with clinical outcomes. We used the scores to describe sequential changes in each organ system in FEAST and to identify clusters of participants with differing physiological derangements within FEAST. We then assessed the effect of fluid bolus on subsequent physiological scores, haemoglobin, and acid-base biochemistry in FEAST participants using a linear model accounting for baseline values as the primary outcome measure. The proportion of individuals with larger, more clinically important changes in physiological scores and blood parameters were compared in a secondary analysis. The effect of bolus volume on physiological scores, haemoglobin, and biochemical parameters was assessed in pre-planned exploratory analyses. The combined contribution of the effects of bolus on time to death was assessed in a post-hoc analysis.

We hypothesised that fluid bolus-induced deaths in FEAST would be associated with detectable changes in cardiovascular, neurological, or respiratory function, oxygen carrying capacity, or blood biochemistry. Several scoring systems exist to predict severity and outcome in paediatric intensive care.^15^, ^16^, ^17^, ^18^ However, these existing scores do not enable evaluation of changes in individual organ systems because they combine markers of dysfunction of multiple organ systems. Furthermore, these scores use blood or intensive care variables that are not routinely available in the low-resource settings where FEAST was done. There are no reliable and established methods to quantify the severity of cardiovascular dysfunction, respiratory dysfunction, or raised intracranial pressure, outside of an intensive care setting. To analyse the effects of fluid bolus on physiological status of each organ system, we developed composite scores to describe respiratory, cardiovascular, and neurological function. We assessed their validity by comparison and analysis of their relationships in all available data from FEAST and four other cohorts of ill children, and then used the composite scores to identify differences in respiratory, neurological, and cardiovascular function between the bolus and no bolus groups of FEAST. We also compared changes in acid-base balance and haemoglobin concentration in bolus and no bolus groups of FEAST. The overall structure of the study and the pre-planned and post-hoc analyses are shown in figure 1.

### Participants

We analysed data from FEAST and two published paediatric studies of critically ill children (meningococcal disease in the UK, and cerebral malaria in Malawi)^3^, ^19^, ^20^ together with data from two unpublished cohorts (one from South Africa, the other from St Mary's Hospital emergency department in London, UK).

In the FEAST study,^3^ between Jan 13, 2009, and Jan 13, 2011, 3170 patients with fever, respiratory distress, or prostration and impaired perfusion were enrolled. 3141 were randomly assigned to either maintenance fluids only (4 mL/kg per h; n=1044), or to receive boluses of 20 mL/kg albumin (n=1050) or normal saline (n=1047) in the first hour, with a further 20 mL/kg if signs of impaired perfusion persisted.^3^ Clinical details of patients in FEAST have been described in detail.^3^, ^21^, ^22^ Vital signs were assessed in surviving children at 1 h, 4 h, 8 h, 24 h, and 48 h after commencing fluid bolus in the albumin and saline groups, or after randomisation in the control group (appendix p 21).

In the UK meningococcal disease study,^19^ 148 fatal cases and 354 survivors with meningococcal sepsis in England and Wales were enrolled between Dec 1, 1997, and Feb 28, 1999. The cohort has been described previously.^19^

In the Malawian cerebral malaria cohort, between Jan 4, 2010, and June 5, 2011, 448 children with cerebral malaria were admitted to Queen Elizabeth Central Hospital, Blantyre, Malawi, and reported in a study of raised intracranial pressure.^20^ Vital signs and clinical variables recorded on admission were related to outcome.

In the South African cohort, 61 children receiving fluid resuscitation for presumed sepsis and gastroenteritis at Red Cross Children's Hospital, Cape Town, South Africa were recruited between Feb 1, 2013, and June 1, 2013. Vital signs and other clinical details were recorded on admission to hospital and related to outcome (admission to intensive care or death).

In the St Mary's cohort, vital sign data and outcome (the need for hospital admission or intensive care admission) were collected for 18 863 children attending the emergency department at St Mary's Hospital, London, UK, from June 10, 2014, to March 9, 2015.

All studies were approved by the institutional review board of the relevant centre. Details of the recruitment procedure, consent procedures, ethics, and institutional approvals for the FEAST, meningococcal, and Malawian cerebral malaria studies have been reported.^3^, ^19^, ^20^ Access to the FEAST trial data was provided after a formal request to the trial study group. Data collection for the South African sepsis cohort and St Mary's Hospital cohort were approved by the local research ethics committee of University of Cape Town (HREC REF 025/2013) and UK (14/LO/0266), respectively; in accordance with these approvals, written informed consent was obtained only for participants who had additional data or samples collected beyond those necessary for routine clinical care. The data from each study were made available with approval of the individual country investigators. The analysis of factors affecting outcome in each study were covered by the ethics approval of each study.

### Outcomes

We analysed the distribution of respiratory, cardiovascular, and neurological function scores among FEAST participants and within each of the four cohorts, and for FEAST compared scores between those who received bolus and those who did not. We used logistic regression to calculate the odds of adverse outcome (death in FEAST and the UK meningococcal disease cohort, neurological sequelae or death in the Malawian cohort, intensive care admission or death in the South African cohort, and admission to hospital or intensive care admission in the St Mary's cohort) for each ten-unit increase in baseline score. Within FEAST participants, we examined the effect of bolus on the proportion of patients with extreme derangement of vital sign scores or physiological parameters, and assessed the effect of volume of fluid administered. Finally, we used Bayesian analysis to identify subgroups with differing physiological derangement who might differ in response to fluids, and to explore how the multiple simultaneous physiological changes caused by fluid bolus were associated with excess deaths in bolus recipients.

### Procedures

The physiological basis and development of composite scores for respiratory function, cardiovascular function, and detection of raised intracranial pressure and neurological function are described in detail in the web appendix (pp 3–6).

To assess respiratory compromise we used respiratory rate (which increases as respiratory function worsens) and oxygen saturation (which decreases as respiratory function worsens). Vital signs were normalised as described in the appendix, with published normal values. We converted age-adjusted degree of tachypnoea and pulse oximetry measurements to the same direction of effect and weighted the contribution of oxygen saturation:

Respiratory score=(respiratory rate – mean respiratory rate for age) + 5 × (100 – oxygen saturation)

Cardiovascular responses to developing shock maintain blood pressure with vasoconstriction and increasing heart rate. If these fail, blood pressure falls. We combined age-adjusted degree of tachycardia, age-adjusted degree of hypotension, and weighted peripheral capillary refill time into a measure of cardiovascular compromise:

Cardiovascular score=(heart rate – mean heart rate for age) + (mean systolic blood pressure for age –systolic blood pressure) + 25 × (capillary refill time)

Raised intracranial pressure is associated with decreasing conscious level, bradycardia, and hypertension (the latter two are components of Cushing's triad).^23^, ^24^, ^25^ We combined weighted level of consciousness measured on the AVPU scale (alert=0; responds to verbal stimulus=1; responds to painful stimulus=2; unresponsive=3),^26^ and age-adjusted degree of hypertension, and age-adjusted degree of bradycardia:

Neurological score=(blood pressure – mean blood pressure for age) + (mean heart rate for age – heart rate) + 25 × (AVPU coma scale)

In FEAST, scores were calculated at baseline (before commencing fluid administration) and 1 h, 4 h, 8 h, 24 h, and 48 h after commencing fluid bolus or maintenance fluids. In the other cohorts, only baseline data (ie, at enrolment) were used.

In FEAST, haemoglobin concentration and plasma lactate were measured at baseline, 8 h, and 24 h; base excess, pH, and electrolytes were measured at baseline and 24 h.^3^

### Statistical analysis

All available data in each dataset were used (appendix pp 21, 22), with the exception of implausible blood biochemistry values in FEAST, which were excluded (base excess less than −30 mEq/L [n=2]; chloride <80 mmol/L [n=1]; bicarbonate=0 mmol/L [n=1]).

We calculated the odds of adverse outcome for each ten-unit increase in baseline score using logistic regression. In FEAST and the UK meningococcal disease cohorts the outcome was death; in the Malawi cohort the outcome was neurological sequelae or death; in the South African cohort the outcome was admission to intensive care, or death; in the St Mary's Hospital cohort the outcome was admission to hospital or intensive care.

To show the association of each physiological score with disease severity, and to gain insight into the association of the scores with specific pathophysiological phenotypes such as cerebral malaria and septic shock, we compared the distribution of scores between cohorts using the Mann-Whitney test.

Saline and albumin bolus were equally associated with increase in mortality in FEAST.^3^ Therefore we compared physiological scores and blood parameters between the combined group of albumin and saline-randomised individuals with those randomised to no bolus in an intention-to-treat analysis. For the primary analysis we used linear regression, controlling for baseline values (before starting fluid), to detect mean differences in physiological scores and blood variables between the bolus and no bolus groups at sequential timepoints after the start of fluid bolus or maintenance fluid (no bolus group) administration.

To examine whether bolus was associated with an increased proportion of patients with extreme derangement of physiological parameters we created separate bins across the distribution of each variable, and then calculated the relative risk for bolus recipients versus no bolus recipients being in each bin. We did this for both change from baseline value, and for absolute values of each parameter.

To assess the effect of volume of fluid administered, we did a secondary analysis based on the actual volume of fluid boluses received within the first 2 h. On the basis of the bimodal distribution, we categorised low volume as less than 30 mL/kg and high volume as 30 mL/kg or more. Because the decision to administer more than 20 mL/kg (initial bolus) was not randomised but was based on clinical assessment of ongoing impaired perfusion, we anticipated some bias due to severity of illness at baseline. We therefore analysed both change from baseline of each physiological score, biochemical variable, and haemoglobin, as well as the actual distribution observed in high-volume and low-volume bolus groups.

To identify subgroups with differing physiological derangement who might differ in response to fluids, we used Bayesian Dirichlet process clustering as implemented in the R package PreMiuM,^27^ applied to baseline physiological scores and haemoglobin concentration and lactate measurements in all participants recruited to FEAST (appendix pp 6, 7). The modelling assumes variables are distributed as multivariate normal distributions within each cluster. We applied Cox proportional hazards regression to each identified cluster to compare survival times between the bolus and no bolus groups. In FEAST stratum A, we used Kaplan-Meier plots to visualise survival over time. We used Spearman correlation to assess the relationship between haemoglobin and lactate.

To explore how the multiple simultaneous physiological changes caused by fluid bolus were associated with excess deaths in bolus recipients (the primary finding in FEAST) we examined the relationship between death and the combination of physiological scores, base excess, bicarbonate, chloride, and haemoglobin concentration in principal component analysis and Cox proportional hazard regression models (appendix pp 6–8). We built Cox proportional hazard models for time of death using bolus or no bolus, physiological scores, and imputed estimates of haemoglobin concentration and biochemical values at 1 h after bolus administration (appendix pp 7, 8). Full details of the imputation and basis for minimal and larger estimates of each value are shown in the appendix (pp 7, 8, 19, 28–31). The initial model included only bolus alone and additional covariates were added iteratively to the models in order of their association with outcome using a forward selection approach. After each iteration, we report the resulting hazard ratio for time of death. We repeated the procedure using the individual components of each physiological score rather than the composite scores, to identify the contribution of each variable to the increased mortality in bolus recipients. For comparison, we also built a multivariable model using measured baseline values of physiological scores and blood parameters. All statistical analyses were done in R (version 3.2.2).

The FEAST trial is registered with ISRCTN, number ISRCTN69856593.

### Role of the funding source

The funders of the study had no role in study design, data collection, data analysis, data interpretation, or writing of the report. The corresponding author had full access to all the data in the study and had final responsibility for the decision to submit for publication.

## Results

Details of each cohort are shown in table 1. In the FEAST study, median physiological scores were 105 (IQR 86–124) for cardiovascular function, 53 (37–79) for respiratory function, and 25 (8–47) for neurological function. Patients in FEAST had significantly higher values of all physiological scores than the St Mary's Hospital cohort (57 [IQR 50–67] for cardiovascular function, 5 (0–12) for respiratory function, and 11 (3–20) for neurological function), and they had higher respiratory scores than all other cohorts (p<0·0001; appendix p 9). Neurological scores were highest in the Malawian cerebral malaria cohort (median score 75, IQR 55–78; appendix p 9). FEAST patients had cardiovascular scores similar to those in the Gram-negative sepsis meningococcal cohort (median score 100, IQR 69–157; appendix p 9).

**Table 1:** Clinical details of cohorts studied

	**Setting**	**Total (n)**	**Entry criteria**	**Age (months)**		**Sex**		**Outcome**		
				n	Median (IQR)	n	Male, n (%)	n	Adverse outcome	Adverse outcome, n (%)
FEAST	Kenya, Uganda, and Tanzania	3170	Fever and respiratory distress or prostration and impaired perfusion	3170	24 (13–38)	3170	1705 (54)	3170	Death	315 (10)
Meningococcal disease	England, Wales, and Northern Ireland, UK	502	*Neisseria meningitidis* culture or PCR positive or purpura fulminans without other cause	499	39 (13–166)	365	192 (53)	502	Death	148 (29)
Cerebral malaria	Blantyre, Malawi	448	Blantyre coma score ≤2 with *Plasmodium falciparum* parasitaemia and no other discernible cause of coma	448	45 (29–67)	448	233 (52)	448	Death or neurological sequelae	106 (24)
South Africa	Cape Town, South Africa	61	Suspected septic shock or severe gastroenteritis and clinician's decision to give fluid resuscitation	61	5 (1–9)	61	36 (59)	61	Death or intensive care admission	20 (33)
St Mary's Hospital emergency department	London, UK	18 863	Emergency department presentation	18 862	48 (18–110)	18 861	10 467 (55)	18 717	Admission to hospital ward or intensive care unit	1933 (10)

Physiological scores in FEAST were highest in the first hours following admission, and decreased over the next 48 h (appendix p 9). Median physiological scores, at baseline and 48 h, respectively, were 105 (IQR 86–124) and 52 (39–65) for cardiovascular score, 53 (37–79) and 18 (11–27) for respiratory score, and 25 (8–47) and 4 (0–13) for neurological score. Patients who subsequently died had significantly higher respiratory and neurological scores than survivors at all timepoints up to 24 h (appendix p 9). Cardiovascular score was significantly higher in fatal cases than in survivors at baseline, was not significantly different at 1 h after administration of initial fluid bolus (if received), but was then significantly higher after 4 h or more in those dying than in survivors (appendix p 9).

In FEAST, odds of death for each ten-unit increase in baseline score was 1·09 (95% CI 1·07–1·11) for respiratory score, 1·26 (1·21–1·31) for neurological score, and 1·09 (95% CI 1·05-1·14) for cardiovascular score (all p<0·0001; appendix p 22). The associations between physiological scores and outcome measures in the other cohorts are shown in the appendix (p 22).

To determine the effect of fluid bolus on physiological scores, biochemical parameters, and haemoglobin concentration, we used linear regression analysis to calculate the mean differences, adjusted for baseline value, between those randomly assigned to receive bolus or no bolus in FEAST. We combined the albumin and saline groups because they had similar effects on mortality in FEAST.^3^ Mean respiratory score was 3·45 (95% CI 0·90–6·01; p=0·0080) higher at 1 h and 2·3 (0·31–4·3; p=0·024) higher at 4 h in children randomly assigned to receive bolus than in those who received no bolus, but there was no significant difference between groups at 12 h (table 2). Fluid bolus increased the mean neurological score at 1 h by 2·64 (95% CI 0·76–4·52, p=0·0060) and there was no significant difference at 4 h and 12 h (table 2). Conversely, fluid bolus decreased the mean cardiovascular score at 1 h by 2·17 (95% CI 0·57–3·78, p=0·0080; table 2); there was no significant difference between groups at 4 h and 12 h. After 4 h, there were no significant differences in physiological scores (table 2). In a post-hoc analysis, we analysed albumin and saline groups separately (appendix p 23). With the exception of respiratory score at 8 h, which was more increased in albumin recipients than in saline recipients, there were no significant differences between participants who received saline and those who received albumin (appendix p 23). Bolus decreased mean haemoglobin concentration at 8 h by 0·33 g/dL (95% CI 0·20–0·46, p<0·0001). Bolus was not associated with a change in blood lactate concentrations at 8 h or 24 h but was associated with a decrease in mean plasma bicarbonate by 0·96 mmol/L (0·45 to 1·47, p=0·0003), a decrease in mean base excess by 1·41 mEq/L (0·76 to 2·06, p<0·0001), and an increase in mean chloride by 1·65 mmol/L (95% CI 0·47 to 2·83, p=0·0070) in patients surviving to 24 h (table 2).

**Table 2:** Changes in physiological scores and blood parameters associated with fluid bolus in FEAST

	**n/N (%)**	Effect size*(95% CI); p value
**Respiratory score**		
1 h	2925/3102 (94·3%)	3·45 (0·90 to 6·01); p=0·0080
4 h	2884/3025 (95·3%)	2·31 (0·31 to 4·31); p=0·024
12 h	2838/2938 (96·6%)	1·58 (−0·29 to 3·46); p=0·10
**Neurology score**		
1 h	2992/3102 (96·5%)	2·64 (0·76 to 4·52); p=0·0060
4 h	2933/3025 (97·0%)	1·05 (−0·68 to 2·79); p=0·23
12 h	2881/2938 (98·1%)	0·322 (−1·30 to 1·94); p=0·70
**Cardiovascular score**		
1 h	3010/3102 (97·0%)	−2·17 (−3·78 to −0·57); p=0·0080
4 h	2948/3025 (97·5%)	−0·30 (−1·93 to 1·33); p=0·72
12 h	2897/2938 (98·6%)	0·10 (−1·52 to 1·72); p=0·90
**Haemoglobin (g/dL)**		
8 h	2771/2974 (93·2%)	−0·33 (−0·46 to −0·20); p<0·0001
24 h	2723/2882 (94·5%)	−0·21 (−0·35 to −0·08); p=0·0010
**Lactate (mmol/L)**		
8 h	2737/2974 (92·0%)	−0·12 (−0·33 to 0·092); p=0·27
24 h	2657/2882 (92·2%)	−0·011 (−0·21 to 0·19); p=0·92
**Base excess (mEq/L)**		
24 h	857/2882 (29·7%)	−1·41 (−2·06 to −0·76); p<0·0001
**Bicarbonate (mmol/L)**		
24 h	858/2882 (29·8%)	−0·96 (−1·47 to −0·45); p=0·0003
**Chloride (mmol/L)**		
24 h	840/2882 (29·1%)	1·65 (0·47 to 2·83); p=0·0070

Results of linear regression analysis comparing albumin or saline bolus with no bolus controls, controlling for respective baseline levels. Times shown are the hours after start of infusion. Proportions are given as a percentage of individuals with data from those alive at that timepoint.

* Mean change in the variable relative to the no bolus control. A positive effect size indicates an increase in the parameter in bolus recipients; a negative effect size indicates a decrease in the parameter in bolus recipients, relative to the no bolus group.

**Figure 2:**
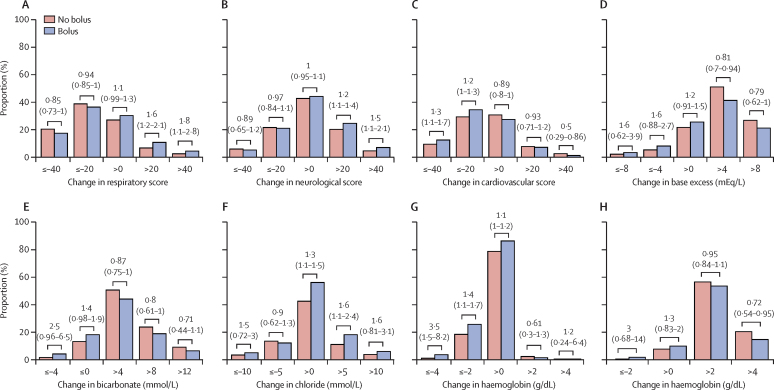
Changes in physiological scores and blood measures associated with fluid bolus The proportion of individuals in FEAST with different magnitudes of change from baseline in physiological scores and blood measurements are shown according to whether they were randomly assigned to receive no fluid bolus (red bars) or fluid bolus (blue bars). Panels A–C show changes in physiological scores between baseline and 1 h after starting fluid infusion. Panels D–F show changes in biochemical measures from baseline to 24 h. Panels G and H show change in haemoglobin concentration from baseline to 8 h in non-transfused (G) and transfused (H) participants. Negative values indicate decrease from the baseline, and positive values indicate increase from baseline. Values above the bars show relative risk (95% CI) for comparison of proportions between bolus and no bolus groups.

To assess whether bolus induced larger changes in some individuals, we compared the proportions of individuals in different segments of the distribution of each variable between the bolus and no bolus groups (figure 2; appendix p 10). Compared with those who did not receive bolus, individuals who received bolus had an increased risk of a large or very large increase in respiratory score and neurological score at 1 h, and of a large or very large decrease in cardiovascular score at 1 h (figure 2). The bolus-associated changes in respiratory score persisted at 4 h (appendix p 10). When absolute values of scores at 1 h and 4 h were considered, rather than change from baseline, a greater proportion of the bolus group had high respiratory and neurological scores than in the no bolus group, whereas a smaller proportion had high cardiovascular score (appendix pp 11, 12). Larger decreases from baseline in base excess and bicarbonate, and larger increases in chloride were observed in bolus recipients (figure 2D–F). A greater proportion of bolus recipients had very low haemoglobin concentration (<7·5 g/dL and <5 g/dL) at 8 h (appendix p 11).

In an exploratory analysis, we assessed the effect of low or high volume bolus on the distribution of physiological scores and blood parameters. At 4 h, the first observation after completion of the high volume bolus, there were higher respiratory and neurological scores in patients who received high volume (≥30 mL/kg) bolus (appendix p 13). However, the distribution of cardiovascular scores at 4 h was similar in low and high volume recipients (appendix p 13). There were lower base excess and bicarbonate, higher chloride, and lower haemoglobin concentration (but only in untransfused participants) in the high-volume bolus recipients (appendix p 13). Findings were similar when change from baseline was considered (appendix p 14).

The biochemical changes we observed in FEAST suggested that boluses of albumin or saline could cause hyperchloraemic metabolic acidosis. To explore this further, we did a post-hoc analysis of the effect of bolus on pH and respiratory compensation. 719 (35%) of 2082 FEAST participants with pH measurements at baseline had acidosis (pH<7·35; appendix p 15), and the mortality rate for those with baseline acidosis was much higher than for those with pH of 7·35 or higher. Among survivors in the bolus group, the relative risk of being acidotic at 24 h was 1·4 (95% CI 1·0–1·9) compared with the no bolus group. There was a significant negative correlation between chloride and pH, with a stronger effect of chloride on pH in the bolus group (appendix p15). Low pH suggests that respiratory compensation mechanisms have been overwhelmed, so we explored the respiratory response at early timepoints, when the majority of deaths occurred in acidotic patients. Respiratory rates were increased at baseline and 1 h, relative to normal ranges for healthy children (appendix p15). Oxygen saturations decreased more in bolus recipients at 1 h, whereas there were no differences between the bolus and no bolus groups in the change in respiratory rate (appendix p 15).

**Figure 3:**
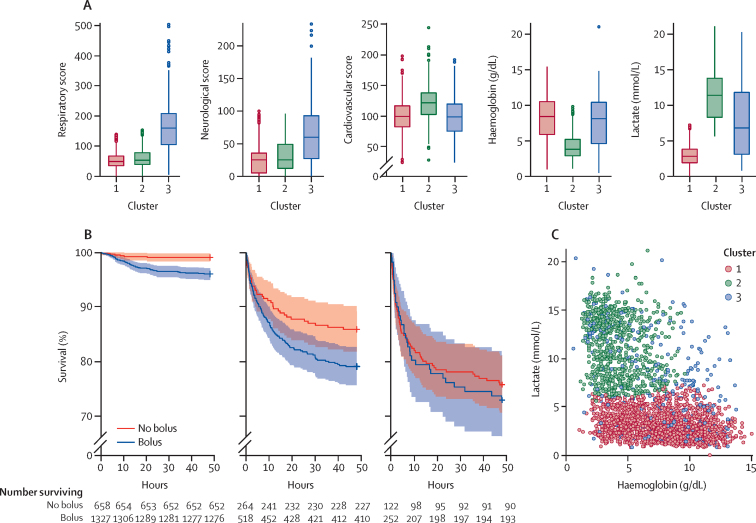
Bayesian cluster analysis of FEAST (A) Distributions of the physiological scores, haemoglobin concentrations, and lactate concentrations by cluster. Boxes show median (coloured line) and IQR; whiskers extend up to 1·5 times IQR. Cluster one, n=1991; cluster two, n=795; cluster three, n=384. (B) Survival curves for no bolus (red line) or bolus (blue line) recipients in each cluster in FEAST stratum A. Cluster one, bolus n=1327, no bolus n= 658; cluster two, bolus n=518, no bolus n=264; cluster three, bolus n=252, no bolus n=122. Dotted lines indicate 95% CIs. (C) Correlation between baseline haemoglobin and lactate concentrations (Spearman p<0·0001, *r*_s_–0·56) with individual participants coloured by cluster (cluster one [red] n=1991; cluster two [green] n=795; cluster three [blue] n=384).

We identified three clusters of patients on the basis of their baseline characteristics (figure 3A). Cluster one (n=1991) had least derangement in physiological scores, haemoglobin concentration, and lactate. Cluster two (n=795) comprised patients with severe anaemia (haemoglobin concentration <5 g/dL), and high lactate and cardiovascular score. Cluster two also had greater base deficit, lower bicarbonate, and high chloride concentrations (appendix p 17). Cluster three (n=384) was characterised by extremely high respiratory and neurological scores, but better maintained haemoglobin concentration.

The clusters differed markedly in mortality (figure 3B). In cluster one, six (1%) of 658 participants in the non-bolus group died, and 51 (4%) of 1327 died in the bolus group. In cluster two, 37 (14%) of 264 patients in the non-bolus group died, and 108 (21%) of 518 died in the bolus group. Mortality was highest in cluster three: 32 (26%) of 122 participants in the non-bolus group died, and 59 (23%) of 252 in the bolus group died.

Considering all participants, there was a negative correlation between haemoglobin and lactate concentrations (Spearman *r*=–0·56, p<0·0001; figure 3C). Associations between bolus and respiratory score, neurological score, haemoglobin concentration, and blood biochemistry in clusters one and two were consistent with those seen in FEAST overall (appendix pp 24–26). The association between bolus and changes in blood parameters and physiological scores in each cluster are shown in the appendix (pp 25–27).

**Figure 4:**
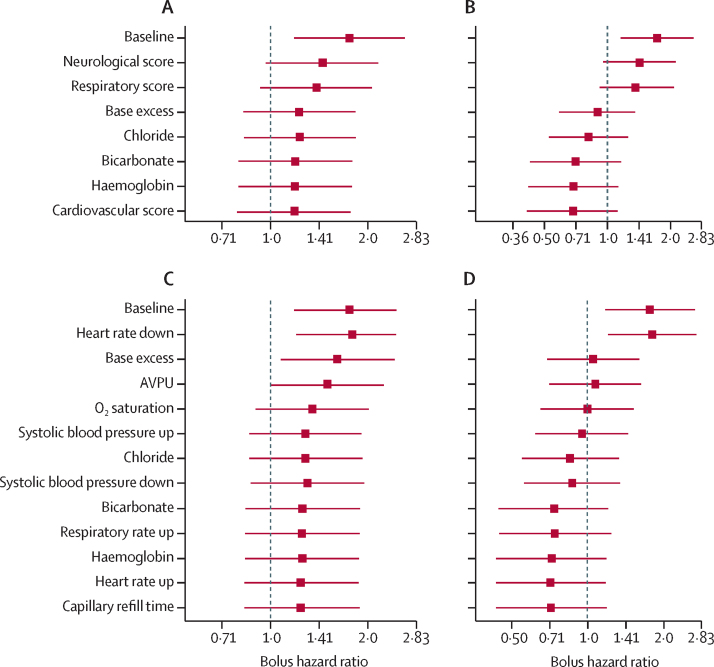
Contribution of physiological derangements to excess mortality due to bolus In post-hoc analyses, we calculated hazard ratios for bolus versus no bolus on time of death in Cox proportional hazard models, sequentially incorporating additional explanatory covariates (in order from top to bottom of each list), showing how the effect of bolus on death is mediated by observed changes in physiology and blood parameters (A–D). Up or down refers to the direction of change from the age-related mean values, which contribute to different physiological scores. (A) The covariate list includes physiological scores at 1 h and conservative (data-derived) estimates of the effect of bolus on blood parameters at 1 h. (B) The covariate list uses literature-derived values for the changes in acid-base biochemistry parameters. (C) The covariate list includes the component variables of the physiological scores at 1 h and conservative (data-derived) estimates of the effect of bolus on blood parameters at 1 h. (D) The covariate list uses literature-derived values for the changes in acid-base biochemistry parameters. Analyses in A–D are based on 1898 subjects with complete data for physiological scores at 1 h and baseline biochemical parameters. AVPU= Alert, Responds to Voice, Responds to Pain, Unresponsive.

**Figure 5:**
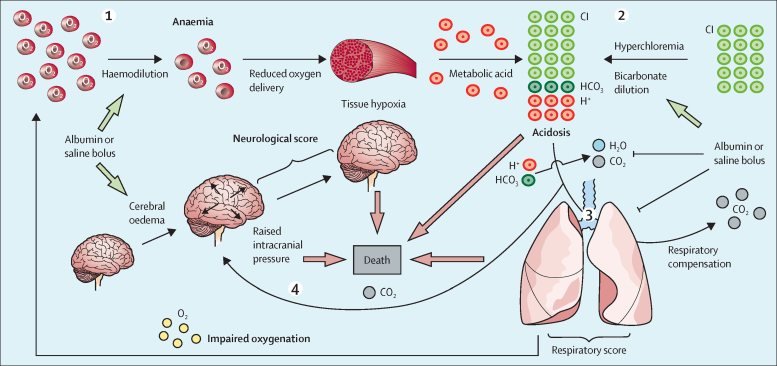
Proposed physiological model of the adverse effects of fluid bolus Bolus fluid reduces haemoglobin concentration, resulting in decreased tissue oxygenation, increasing anaerobic metabolism, and metabolic acidosis. According to the Stewart model, maintenance of normal plasma pH is controlled by (1) the strong ion difference (charge difference between strong cations (Na^+^, K^+^, Ca^2+^, and Mg2+), and strong anions (Cl– and lactate–); (2) pCO_2_ and (3) charge from weak acids (phosphate, albumin).^28^, ^29^ Bolus of normal saline or 5% albumin (which have similar electrolyte content) caused hyperchloraemia and dilution of bicarbonate, resulting in a reduction in the strong ion difference. Hyperchloraemic acidosis increases the need for respiratory compensation through increased carbon dioxide excretion to maintain pH. Worsening of respiratory function due to bolus results in hypoxia (as evidenced by low oxygen saturation and increased respiratory score). This outcome, together with an inability to increase respiratory rate, impairs excretion of carbon dioxide (not shown in our study). Increasing carbon dioxide causes cerebral vasodilation, resulting in increased intracranial pressure. Fluid bolus might also directly cause cerebral oedema. The combination of adverse effects on haemoglobin concentration, acidosis, and respiratory and neurological function induced by modest albumin or saline fluid boluses might overwhelm compensatory mechanisms in the most severely ill patients, resulting in increased mortality.

We reasoned that the multiple physiological changes induced by bolus might act in combination to explain the higher mortality observed in the bolus group, and investigated this in post-hoc analyses. Principal component analysis using the physiological scores and predicted values of blood parameters at 1 h showed that the distribution of fatal cases was distinct from survivors and appeared related to the effects of bolus (appendix p 18). Baseline values of these variables predicted deaths in both bolus and no bolus groups, but there were more observed deaths in the bolus group, indicating that the baseline values of the covariates did not explain the difference in mortality (as expected in a randomised trial; appendix p 32). However, when we used the 1 h post-bolus values to predict deaths, using either conservative or more realistic estimates of the biochemical changes induced by bolus at 1 hour (appendix pp 7, 8, 28–32), we found that there was no longer a significant difference between the bolus and no bolus groups (figure 4; appendix p 32). This finding indicates that the covariates in the model can explain all of the difference in observed mortality between bolus and no bolus groups of the trial. We found that after bolus, the neurological score, base excess, and respiratory score were the major determinants of the increased death rate from bolus using either minimal (figure 4A) or more realistic estimates (figure 4B) of the post-bolus biochemical values (appendix). When the individual components of each score were included in the models, the post-bolus AVPU score, base excess, and oxygen saturation made the largest contribution to explaining the excess deaths (figure 4C, 4D). We summarised these findings into a physiological model proposing how the adverse effects of bolus fluids could increase mortality (figure 5).

## Discussion

The physiological scores we developed provide a tool to quantify changes in respiratory, cardiovascular, and neurological function in response to bolus fluids in FEAST. The scores discriminated between cohorts of patients with infections affecting different organ systems and were generally predictive of outcome, showing their use as objective measures of organ system derangement. Using these physiological scores and available blood measurements, we observed six deleterious effects of fluid bolus: respiratory and neurological scores increased at early timepoints; haemoglobin concentration decreased (particularly in patients who did not receive blood transfusion); base deficit and chloride concentrations increased, and plasma bicarbonate decreased (changes consistent with hyperchloraemic acidosis). Although the linear regression analysis showed a modest, but significant, change in the mean value of each variable, there were much greater changes observed in some individuals, with increased proportions of bolus recipients having extreme adverse physiological derangement in physiological scores, haemoglobin concentration, and blood chemistry. Most of these changes were greater in patients receiving higher fluid volumes. The changes in physiological scores were most evident at early timepoints, but early deaths and the increased mortality in bolus recipients makes evaluation of later timepoints difficult. The only beneficial physiological change observed was a reduction of cardiovascular score, in keeping with the impression of clinicians that perfusion improved during fluid resuscitation.

When we included the bolus-induced changes in respiratory function, cardiovascular function, neurological function, and biochemical changes in Cox survival models, the increased mortality in bolus recipients in FEAST was explained by the physiological changes we identified. Analysis of the effect of each component in the models showed that the excess deaths in bolus recipients were largely explained by worsening neurological score (or raised intracranial pressure), increased base deficit, and worsening respiratory score. Of the individual components analysed, bolus-induced increased base deficit, worsening conscious level, and oxygen saturation were the major contributors to the excess mortality associated with bolus.

Since the 1990s, there have been increasing reports, including small case studies, randomised trials in healthy volunteers and patients, and experimental studies in animals that have documented hyperchloraemic acidosis after infusion of unbuffered salt solutions (appendix pp 28–31).^30^, ^31^, ^32^, ^33^ Hyperchloraemic acidosis is an inevitable consequence of the infusion of unbuffered sodium chloride solutions, predicted by the Stewart equation of acid-base balance^28^, ^29^ and confirmed in the extensive literature. Although many small studies comparing balanced salt solutions (which contain acetate or lactate as a source of anion) with normal saline have documented saline-induced hyperchloraemic acidosis, recent large clinical trials also reported increased mortality and worsening renal outcome in adults receiving normal saline as compared with balanced salt solutions.^34^, ^35^ Our finding that the bolus-induced hyperchloraemic acidosis and decreased base excess was a major contributor to the increased mortality in bolus recipients suggests that much of the adverse effect of bolus in FEAST was due to the nature of the fluids used.

Worsening respiratory function and increase in signs of raised intracranial pressure, which we documented in bolus recipients, are well known effects of fluid administration in some critically ill patients. Indeed, many guidelines for management of shock recommend intubation, and elective mechanical positive pressure ventilation for patients who remain with signs of shock after initial bolus volume infusion because of the recognition that continued fluid resuscitation is likely to cause respiratory deterioration.^1^, ^36^ However, our finding that small volumes (20–40 mL/kg) of normal saline or albumin, which are routinely administered to underperfused children in emergency departments, cause worsening of respiratory function and decrease in oxygen saturation suggest that even modest volumes of fluid might have detrimental effects in some children despite the observable improvement in perfusion, which was also manifested in FEAST as improving cardiovascular score. Our findings raise the possibility that administration of fluid bolus to underperfused patients might increase the need for intensive care admission because of worsening oxygenation.

The worsening neurological score in bolus recipients and the contribution of deteriorating neurological score to the excess mortality in bolus recipients is also an expected effect of bolus fluids in patients who have increased intracranial pressure. More than 50% of the patients included in FEAST had severe malaria, and many of these patients had evidence of cerebral malaria. Evidence of raised intracranial pressure has been well documented in cerebral malaria by direct measurement,^37^ MRI scan,^20^ and by clinical findings.^38^ Both fluid volume and the hyperchloraemic acidosis induced by the saline and albumin used in FEAST could have contributed to the worsening of consciousness and neurological score in bolus recipients (figure 5). Although patients with neurological diseases such as cerebral malaria and meningitis comprise a smaller proportion of critical illness in resource-rich settings, our finding that worsening neurological score occurred in bolus recipients suggests that volume resuscitation should be used with great caution in patients presenting with neurological illness, and unbuffered salt solutions should be avoided.

Our cluster analysis revealed heterogeneity within FEAST. The majority of patients (cluster one), had least severe derangement of all three physiological scores, adequate haemoglobin concentrations, and remarkably low mortality in the absence of bolus. Fluid bolus in this cluster caused worsening of respiratory and neurological function, reduced haemoglobin concentration and increased acidosis. The second cluster, characterised by low haemoglobin concentration, high lactate concentration, and high cardiovascular score, identified patients with severe anaemia. The inverse relationship observed between haemoglobin and lactate concentrations, and lower bicarbonate and base excess suggests that the baseline hyperlactataemia and metabolic acidosis is largely driven by impaired oxygen carrying capacity. In this cluster, bolus fluids caused worsening of respiratory and neurological function and increased acidosis. The third cluster, characterised by high respiratory and neurological scores had high mortality (23–26%). Patients in the bolus group had lower haemoglobin concentrations and higher chloride concentrations than those in the non-bolus group.

Identification of clusters within the large FEAST cohort of ill children has important therapeutic implications. The low mortality in the absence of bolus fluids in cluster one suggests that this group did not require any additional intervention, and most would recover with treatment of the infection alone. In cluster two, in which anaemia was the major problem, we believe blood transfusion was required, and bolus probably worsened tissue oxygen delivery. In cluster three, in which respiratory failure and neurological impairment were the main physiological derangements, we believe that respiratory support was needed to reduce mortality.

Combination of the physiological derangements caused by bolus explained mortality better than the pre-bolus values of the same parameters, indicating that through their combined effects, the bolus-induced changes plausibly explain the effect of bolus on mortality in FEAST (figure 4). Because the physiological changes identified are likely to be similar in all settings, our findings have implications for fluid resuscitation in both developed and resource-limited settings, although the magnitude of effect may differ because the severity of anaemia in FEAST participants might have increased the relative importance of haemodilution.

The findings in our analysis differ from the previous analysis of modes of death in FEAST^9^, ^22^ and an ovine model,^39^ which concluded that worsening cardiovascular shock was the major adverse effect of bolus. The earlier study^22^ used increased plasma lactate concentration as part of the definition of cardiovascular shock. Our analysis found that plasma lactate was negatively correlated with haemoglobin concentration, and thus use of lactate as a biomarker of cardiovascular shock in the previous analysis gave an incorrect indication of the prevalence of cardiovascular shock.

Our study has several limitations. Although our physiological scores are associated with outcome in multiple different cohorts, they have not been independently validated. In particular, the weightings were chosen to reflect the perceived importance of each variable as a measure of organ system function. They were not derived from the data or from previous studies. The scores should be seen as means of studying changes in physiology between arms of a trial, rather than as outcome predictors, for which there are already a number of scoring systems available.^15^, ^16^, ^17^, ^18^ FEAST was done in settings in which intensive care and mechanical ventilation were not available. Although the physiological changes induced by bolus are likely to be seen in all settings, caution is needed in extrapolating associations with mortality to resource-rich settings, in which some of the adverse effects of bolus fluids could be mitigated by mechanical ventilation and neuro-intensive care. Furthermore, because of the design of the trial, we cannot exclude that the associations we have reported between greater physiological derangements and larger volumes of administered fluid are confounded by the indication—ie, administration of larger volumes of fluid to participants with more deranged physiology. An additional limitation is that acid-base balance was only measured on admission and at 24 h, by which time most deaths had occurred, and many patients had no data at the 24 h timepoint. To include the effect of bolus on acid-base balance, we imputed the earlier timepoints from the 24 h differences, and used published values to produce a range of estimates of the extent of acidosis at earlier time points. We believe the assumptions used in the imputation provide reliable estimates of the earlier values.

The FEAST trial included children with widely differing diseases and underlying physiology. We found that fluid bolus achieved the intended improvement in cardiovascular function at the cost of worsening respiratory and neurological function, acidosis, and reduced haemoglobin. Our finding that within FEAST there are clusters with differing physiological derangements suggests particular need for caution in fluid administration to patients with established respiratory failure, neurological failure, severe acidosis, or anaemia, and the need for better detection and specific management of the physiological derangements in each individual patient. The physiological scores we have developed might be useful in identifying derangement in specific organ systems.

The combination of adverse physiological effects we observed, associated with both albumin and saline bolus, raises questions about both the volume and type of fluid used in resuscitation. Balanced salt solutions such as Ringer's lactate or plasmalyte (which have electrolyte compositions closer to plasma than normal saline, and lactate or acetate as major anions) are associated with more rapid resolution of acidosis than 0·9% saline, and in recent clinical trials they were associated with improved outcomes.^34^, ^35^, ^40^ Our finding that worsening hyperchloraemic acidosis was a major contributor to the excess deaths in FEAST suggests that unbalanced salt solutions such as normal saline and 5% albumin should be avoided in seriously ill patients and replaced with balanced salt solutions. However, other fluids are likely to have the same adverse effects on respiratory function, oxygen carrying capacity, and perhaps neurological function that we have observed with albumin and saline in FEAST. Trials are needed to establish if balanced electrolyte solutions and lower volumes than currently recommended will allow the benefits of fluid on cardiovascular function to be achieved without the adverse effects observed in FEAST.
