# Supplementary material for: Effects of saline or albumin fluid bolus in resuscitation: evidence from re-analysis of the FEAST trial
Source: Lancet Respir Med. 2019 Jul;7(7):581–93. doi: 10.1016/S2213-2600(19)30114-6 (PMC6593355; doi:10.1016/S2213-2600(19)30114-6)
Supplement: Supplementary appendix [file mmc1.pdf]

THE LANCET
Respiratory Medicine

Supplementary appendix

This appendix formed part of the original submission and has been peer reviewed. We post it as supplied by the authors.

Supplement to: Levin M, Cunnington AJ, Wilson C, et al. Effects of saline or albumin fluid bolus in resuscitation: evidence from re-analysis of the FEAST trial. *Lancet Respir Med* 2019; published online June 10. http://dx.doi.org/10.1016/S2213-2600(19)30114-6.

Web appendix: Adverse effects of saline or albumin fluid bolus in resuscitation: Evidence from re-analysis of the FEAST trial

Table of Contents

List of Investigators 3

Detailed methods 3

Relationship to the FEAST trial and analysis plan 3

Assessment of changes in physiology and blood parameters 3

Developing a composite measure for cardiovascular function 3

Developing a composite measure for respiratory function 5

Developing a composite measure for raised intracranial pressure and neurological status 5

Clustering algorithm 6

Principal component analysis 6

Evaluation of the effect of bolus-induced changes in blood parameters on survival 7

Cox proportional hazards regression modelling 8

Covariate set 1 8

Covariate set 2 8

Covariate set 3 8

Covariate set 4 8

Web extra Figures 9

Web extra Figure 1. Physiological scores associated with aetiology and severity of illness across cohorts and sequential changes over time in FEAST 9

Web extra Figure 2. Change in physiological scores from baseline to 4 hours. 10

Web extra Figure 3. Distribution of physiological scores and blood parameters at first observation after randomization in FEAST. 11

Web extra Figure 4. Distribution of physiological scores at 4 hours in FEAST. 12

Web extra Figure 5. Distribution of physiological scores and blood parameters associated with volume of fluid bolus administered in FEAST. 13

Web extra Figure 6. Changes from baseline to next observation for physiological scores and blood measures according to volume of fluid bolus received in FEAST. 14

Web extra Figure 7. Hyperchloraemic acidosis in FEAST. 15

Web extra Figure 8. Biochemical measurements in clusters within FEAST. 17

Web extra Figure 9. Principle component plot using physiological scores and blood parameters. 18

Web extra Figure 10. Imputation of blood parameters based on relationships between baseline and 24 hour values. 19

Web extra Figure 11. Relationship between baseline values and change at 24 hours for biochemical parameters. 20
Web extra Tables 21
Web extra Table 1. Numbers (%) of surviving subjects at each time point in FEAST Stratum A with available data for each variable. 21
Web extra Table 2. Associations between physiological scores and outcome* in FEAST and 4 other cohorts. 22
Web extra Table 3. Changes in physiological scores and blood parameters associated with albumin bolus or saline bolus in FEAST. 23
Web extra Table 4. Association between changes in variables and risk of death in clusters within FEAST. 24
Web extra Table 5. Changes in physiological scores and blood parameters associated with fluid bolus in FEAST Cluster 1 25
Web extra Table 6. Changes in physiological scores and blood parameters associated with fluid bolus in FEAST Cluster 2. 26
Web extra Table 7. Changes in physiological scores and blood parameters associated with fluid bolus in FEAST Cluster 3. 27
Web extra Table 8. Studies evaluating the effects of unbalanced salt solutions on blood acid-base balance and chloride 28
Web extra Table 9. Cox proportional hazard regression models for time of death. .. 32
References 33

List of Investigators

Michael Levin, Aubrey J. Cunnington, Clare Wilson, Simon Nadel, Hans Joerg Lang, Nelly Ninnis, Mignon McCulloch, Andrew Argent, Heloise Buys, Christopher A. Moxon, Abigail Best, Ruud G. Nijman, and Clive J. Hoggart

Detailed methods

Relationship to the FEAST trial and analysis plan

The present study was not planned as a pre-specified analysis of the FEAST trial. The present study represents a hypothesis-based reanalysis of the trial data. The primary hypothesis was that bolus fluids produce measurable changes in cardiovascular function, respiratory function, raised intracranial pressure or neurological function, oxygen carrying capacity, biochemical and acid-base status. The secondary hypothesis was that if these changes are detectable, they can explain the excess mortality associated with bolus fluid in the FEAST trial. To evaluate both of these hypotheses, multiple analyses (detailed below) were performed in order to justify the methodological approaches for detection of the changes in organ system function and to assess whether any effects attributed to bolus were consistent with expected relationships with the volume of bolus-fluids received, known mechanisms of acid-base derangement, and between subgroups of patients.

Assessment of changes in physiology and blood parameters

FEAST collected data on respiratory rate, pulse rate, blood pressure, capillary refill time and level of consciousness sequentially throughout the trial at baseline, 1, 4, 8, 24 and 48 hours[1]. Haemoglobin and lactate concentrations were measured at baseline, 8 and 24 hours.[1] Plasma chemistry and acid-base balance were measured at baseline and 24 hours.[1]

Our first challenge in attempting to establish the mechanisms by which fluid increased mortality in FEAST was to identify a methodology for detecting changes in each organ system in bolus recipients using the data available from the trial.

There are currently no “gold standard” methods to quantify respiratory function, neurological function or cardiovascular function in critically ill children based on clinical features, although multiple international guidelines indicate that respiratory rate, pulse rate, blood pressure, capillary refill time and level of consciousness should be used to guide management. We postulated that combinations of these variables would provide objective and quantitative indicators of respiratory, neurological and cardiac function, in a similar manner to which experienced clinicians subconsciously assimilate them each time they assess a seriously ill patient**.** As blood pressure, heart rate, and respiratory rate vary greatly in children of different age, we adjusted for age by calculating the deviation from the age-related mean values for healthy children. Mean values for heart rate and respiratory rate and blood pressure were derived from published tables.[2, 3]

We note that the intended purpose of each score was to enable comparison of changes in organ function between the bolus and no bolus arms of the randomised controlled trial, and to compare physiological derangement between different studies. Recognising that there is no absolute measure against which to calibrate each score, the weightings of oxygen saturation, coma score and capillary refill time were chosen to reflect the clinical importance of each component of the score. We acknowledge that different weightings might enable better prediction of outcome in individual studies, however our aim was to develop an objective tool for comparison between arms and studies, and not to develop a new predictor of outcome.

Heart rate and blood pressure deviations from the age-related means were considered to have different implications for cardiovascular and neurological status, since low blood pressure and high heart rate are features of circulatory failure whilst rising blood pressure and falling heart rate are features of raised intracranial pressure. Thus only physiologically adverse deviations contributed to each score, and otherwise the difference was set to zero as explained for each score below.

Developing a composite measure for cardiovascular function

Measurement of cardiac function is routinely undertaken using echocardiography or thermodilution catheter in an intensive care setting. However these methods were not available in FEAST. Furthermore, shock is defined as a life-threatening, generalized form of acute circulatory failure associated with inadequate oxygen utilization by the cells; a state in which the circulation is unable to deliver sufficient oxygen to meet the demands of the tissues resulting in cellular dysfunction.[4] Therefore it is not solely defined by or dependent on cardiac output. Remarkably neither adult nor paediatric guidelines for clinical recognition of shock have an objective quantification of the severity of shock (see table below). In some guidance more severe cases are recognized by being refractory to fluid volume expansion[5] thus defining severity by the very intervention shown to be associated with increased mortality in FEAST. Clinical recognition of shock is based not only on markers of cardiac output (such as heart rate and blood pressure) but on evidence of impaired perfusion of tissues and organs, such as change in mentation,

oliguria or rising blood lactate.[4, 5] Furthermore FEAST included a large proportion of patients with presumed cerebral malaria and meningitis[1, 6] so changes in mentation may not only be caused by impaired cardiovascular function or perfusion but by the underlying disease process. Furthermore, changes in urine output were not recorded as this is difficult without indwelling catheters. Lactate, which is the most commonly used marker of tissue under perfusion in most shock algorithms, was also found to be unreliable in the African setting because we found it was closely correlated with the level of haemoglobin and thus more a reflection of anaemia than cardiovascular perfusion.

Criteria for clinical recognition of shock (modified from Houston et al.[7])

**Guideline**	**Clinical criteria**	Age group
**Advanced Paediatric Life Support (APLS)[8]**	Compensated: normal blood pressure (BP), but capillary refill time (CRT) >2 s, mottled peripheries, peripheral cyanosis<br>Decompensated: as above but with hypotension, decreased mental status	Paediatric
**American Academy of Critical Care Medicine – Paediatric Advanced Life Support (ACCM-PALS)[9]**	Septic shock: Suspected infection (hypo- or hyperthermia) and clinical signs of inadequate perfusion including any of: decreased or altered mental status; CRT >2 s (cold shock) or flash CRT (warm shock), diminished (cold shock) or bounding (warm shock) peripheral pulses, mottled cool extremities (cold shock), or decreased urine output (<1 ml/kg/hr)	Paediatric
**World Health Organization (WHO)[10]**	Shock: cold hands, capillary refill time longer than 3 s, high heart rate with weak pulse, and low or unmeasurable blood pressure	Paediatric
**Fluid Expansion As A Supportive Therapy (FEAST) study[1]**	History of fever and temperature ≥37·.5 °C or <36·.0 °C and impaired consciousness (prostration or coma) and/or respiratory distress<br>Stratum A (impaired perfusion) Plus ≥1 of: CRT >2 s; lower limb temperature gradient; weak pulse; tachycardia (defined)<br>Stratum B (decompensated shock): Systolic BP <50 mmHg if < 12 months old; <60 mmHg if 1–5 years old; <70 mmHg if > 5 years old	Paediatric
**International pediatric sepsis consensus conference definitions[11]**	Sepsis: SIRS (2 of 4 of: core temp > 38·5 or < 36 °C; tachycardia or bradycardia if <1yr; tachypnea; elevated or depressed leukocyte count) in the presence of or as a result of suspected or proven infection<br>Septic shock: Sepsis and cardiovascular organ dysfunction	Paediatric
**Sepsis 3[12]**	Sepsis: life-threatening organ dysfunction caused by a dysregulated host response to infection. Organ dysfunction defined as an increase in the SOFA score of 2 points or more<br>Septic shock: Sepsis and both persistent hypotension requiring vasopressors and lactate ≥2 mmol/L despite adequate volume resuscitation	Adult
**Sepsis 2[13]**	Sepsis: suspected or documented infection with change to some of the following: General variables; inflammatory variables; haemodynamic variables; organ dysfunction variables ; tissue perfusion variables.<br>Severe sepsis: sepsis complicated by organ dysfunction<br>Septic shock: persistent arterial hypotension unexplained by other cause	Adult

These clinical indicators of cardiovascular system dysfunction are rarely synthesised into quantitative measures which would allow comparison of organ function between individuals or sequentially over time. The widely used shock-index was devised based on the logical but arbitrary belief that heart rate divided by systolic blood pressure should provide a better indicator of cardiovascular status than either alone,[14] but was not empirically derived based on data. For application to children it has required modifications to account for age-related variation in normal values,[15] and it does not include any measure of perfusion.

We concluded there was no optimal or accepted method for quantifying differences in shock or cardiovascular function between the bolus and no bolus arms of FEAST, and that we needed to develop novel methods that could be used to identify the effect of bolus on shock /cardiovascular function (as well as for each of the other organ systems, described below) using the available data.

We reasoned that an overall assessment of cardiovascular function could be made by combining heart rate and blood pressure with capillary refill time, which is a well-established marker of perfusion, as all three variables were recorded sequentially in FEAST. Heart rate rises while blood pressure falls as shock evolves, so heart rate and blood pressure can be combined as markers of cardiac function by converting them to the same direction of effect. As all vital signs are age related we also adjusted blood pressure and heart rate to detect a rise in heart rate above the mean normal for age and a fall in blood pressure below the mean normal for age. As capillary refill time is typically in the range of 0 to 5 seconds, while heart rate and blood pressure extend over much larger numerical ranges, we weighted capillary refill time so that it could make an approximately equal contribution to an overall score for cardiovascular function.

The conventional method for assigning weightings in predictive scores is to derive the weightings from the associations between the data and the outcome of interest, and then test the assigned weightings on an independent

and external data set. However, as we were aiming to characterise the changes in organ system function induced by bolus fluids, rather than aiming to identify the best predictor of outcome (death in the case of FEAST), there was no gold standard outcome against which to evaluate the weightings of each variable. The clinical definitions of shock listed above include a range of variables, but provide no method for quantifying the severity of derangement. We concluded that empiric assignment of weighting for capillary refill time to enable a contribution equivalent to that of blood pressure and pulse rate was appropriate. Further evidence on the utility of the score could be gained by comparison of the composite score between cohorts, and evaluation of the weightings can be achieved in modelling the effect of each variable in survival models (see below). The composite score we used was:

Composite cardiovascular score

Cardiovascular score = (heart rate - mean heart rate for age) + (mean blood pressure for age – blood pressure) + 25(capillary refill time)

If the heart rate was less than the mean heart rate for age, the term (heart rate - mean heart rate for age) was set to zero, so that only increased heart rates contributed to the score. If the blood pressure was greater than the mean blood pressure for age, the term (mean blood pressure for age - blood pressure) was set to zero, so that only decreased blood pressure contributed to the score.

Developing a composite measure for respiratory function

Assessment of respiratory function is often achieved by assessment of the adequacy of oxygenation (comparing the ratio of inspired oxygen to arterial oxygen in ventilated patients) and of carbon dioxide excretion. However, accurate evaluation of respiratory function is more difficult in patients who are not mechanically ventilated as widely differing oxygen concentrations are delivered when using face mask or nasal cannula. Clinical assessment of respiratory function in settings other than intensive care units is generally undertaken by observing the rate of breathing, respiratory effort and the depth of breathing and by pulse oximetry. Depth of breathing and respiratory effort are highly subjective and difficult to quantify but respiratory rate and pulse oximetry are reliably measured and were recorded sequentially during the first 48 hours of the FEAST trial. We reasoned that an overall assessment of respiratory function could be made by combining in a composite score, respiratory rate (which rises as respiratory function is compromised) with oxygen saturation (which declines) by converting them to the same direction of effect. As oxygen saturation declines over a more limited range than the changes in respiratory rate and as there may be more clinical significance to a declining oxygen saturation, we weighted contribution of declining oxygen saturation in a combined score.

Composite respiratory score

Respiratory score = (respiratory rate - mean respiratory rate for age) + 5(100 - oxygen saturation)

If the respiratory rate was less than the mean respiratory rate for age, the term (respiratory rate - mean respiratory rate for age) was set to zero, so that only increased respiratory rates contributed to the score.

Developing a composite measure for raised intracranial pressure and neurological status

How to detect raised intracranial pressure (ICP) is the most difficult challenge in evaluating the possible mechanisms for increased death in bolus recipients. The only reliable method for detecting raised intracranial pressure is by directly inserted intracranial pressure transducer, and there is no established method to detect or monitor raised ICP outside an intensive care setting[16]. Detection of raised ICP using MRI or CT scan is notoriously unreliable unless the changes are extremely severe. In practice, suspicion of raised intracranial pressure is raised clinically in patients who have declining levels of consciousness and who also show evidence of brainstem compression of centers controlling respiration, blood pressure and heart rate. Patients who show paradoxical bradycardia, rising blood pressure and declining consciousness or abnormal respiratory patterns (components of Cushing's triad[17-19]) are considered to have features of raised intracranial pressure and generally managed in intensive care units with intracranial monitoring and brain imaging. We postulated that the same clinical variables used by clinicians to detect raised intracranial pressure clinically could be combined in a single score combining rising blood pressure, falling heart rate and declining consciousness. As with the cardiovascular score, we converted the rising blood pressure and falling heart rate to the same direction of effect, and normalized rising blood pressure and falling heart rate using age-related means. As coma was measured by the AVPU (awake (A), response to voice (V), response to pain (P), unresponsive (U)) scale over a much smaller range than blood pressure and heart rate we weighted its contribution in order to capture its contribution to the overall score.

FEAST utilised two different coma scores,[1] at baseline the Blantyre Coma Score[20] was recorded which summates three measures of responsiveness: motor (able to localize=2, withdraws from pain=1; unresponsive=0), eye movement (follows=1; unresponsive=0), and voice (appropriate response=2, inappropriate or groaning=1, unresponsive=0), giving a total score from 0 to 5 which decreases with severity. At later time points the AVPU scale was used. In order to contribute to neurological score the AVPU categories were given numerical values A=0, V=1, P=2, U=3. In order to enable comparison of baseline with later time points we converted baseline Blantyre Coma Score to AVPU as follows:

Blantyre Coma Score	AVPU scale	Numerical score
5	A	0
4	V	1
3	V	1
2	P	2
1	U	3
0	U	3

We weighted the contribution of AVPU to reflect its expected importance as an indicator of impaired neurological function.

Composite neurological score

Neurological score = (blood pressure - mean blood pressure for age) + (mean heart rate for age – heart rate) + 25 (AVPU score).

If the blood pressure was lower than the mean blood pressure for age, the term (blood pressure - mean blood pressure for age) was set to zero, so that only increased blood pressure contributed to the score. If the heart rate was greater than the mean heart rate for age, the term (mean heart rate for age - heart rate) was set to zero, so that only decreased heart rates contributed to the score.

Clustering algorithm

Individuals were clustered using their physiological scores and haemoglobin and lactate measures at baseline. Treatment and outcome were not included in the clustering. Clustering was implemented using a Bayesian multivariate normal mixture of Dirichlet process (MDP) model as implemented in the R package PreMiuM.[21,22] In our implementation each cluster in the mixture is a multivariate normal distribution representing the distribution of the measures within the cluster. An advantage of a MDP model is that the number of clusters is unknown and inferred from the data and inference is made by sampling from the posterior distribution using Markov chain Monte Carlo (MCMC). PreMiuM calculates an optimum single clustering and assignment of individuals to the clusters from the posterior samples it simulates; firstly it calculates a similarity matrix between all pairs of individuals based on the posterior mean of the number of times each pair is assigned to the same cluster. Individuals are then robustly assigned to clusters in a way consistent with the similarity matrix for all possible number of clusters. The optimal number of clusters is determined by the partition which maximises the average silhouette width. We used the single optimal clusters and assignments in all subsequent analyses. The optimal number of clusters determined by the algorithm is driven by the prior distribution assigned to the within cluster covariance; the more prior weight assigned to larger variances the fewer the clusters as more variation is allowed within each cluster. The default settings of the program resulted in eight clusters, increasing the prior within cluster variance by a factor of ten relative to the default values resulted in three clusters which we deemed to be more interpretable and therefore used in subsequent analyses.

Principal component analysis

Principal component analysis (PCA) was performed on the matrix of respiratory, neurological and cardiovascular scores at one hour and baseline base excess, bicarbonate, chloride and haemoglobin in the no bolus group; in the bolus group, to account for changes in the blood parameters which were not measured at one hour, values were adjusted by the observed mean shifts at 24 hours in biochemical variables (base excess -1.41, bicarbonate -0.96, chloride +1.65) and at 8 hours for haemoglobin (-0.32). This represents a conservative estimate of the change in these variables in the bolus group at one hour. Data was standardized such that all covariates had mean zero and standard deviation of one before PCA. Only samples with complete measures for all seven covariates were used, n=1901. The principal component loadings are weights representing the contribution of each of the seven covariates to each principal component. The loadings V are calculated as follows:

$$V = X^T U D^{-1/2}$$

where V are the PC loadings, X is the data matrix, U is the matrix of PCs and D is the diagonal matrix of eigenvalues.

Evaluation of the effect of bolus-induced changes in blood parameters on survival

Evaluation of changes in plasma chemistry and acid-base balance due to bolus

Acid-base balance and plasma chemistry were only measured on admission (before bolus fluids were administered) and again 24 hours later. We therefore had no data on the actual values for plasma chemistry and acid-base status in the early hours of the trial when most of the deaths were occurring. Furthermore by 24 hours, when the second measurement of plasma biochemistry was undertaken, the majority of deaths in the study had already occurred. Therefore we used two approaches to estimate the likely biochemical and acid-base status immediately after bolus-administration. First we used the difference in these parameters between surviving bolus and no bolus recipients at 24 hours to calculate a conservative estimate of the change in these parameters immediately after bolus administration. Second we used a literature-based estimate of the likely magnitude of change due to albumin or saline bolus.

Supporting the first approach, as shown in webextra Figure 10, baseline levels of bicarbonate, chloride and base excess were highly correlated with the levels at 24 hours in surviving patients. However, as shown in webextra Figure 11, patients with more severe derangement of base excess, bicarbonate and chloride at baseline had a greater change between 0 and 24 hours than those with less derangement. We concluded that the linear interpolation of the earlier time points from the 24 hour data was likely to significantly under estimate the change at the earlier time point. Examination of the 24 hour blood sample results (Table 1 in the main manuscript) showed clear evidence of worse hyperchloraemic acidosis in bolus recipients, who had significantly lower plasma bicarbonate and increased base excess and chloride as compared to the no bolus controls. As increased base excess was a strong predictor of death and those patients who went on to die had lower base excess and bicarbonate at baseline than survivors, the 24 hour values available were likely to significantly underestimate the extent of bolus-induced acidosis due to the higher death rate in bolus recipients by 24 hours.

In order to estimate the bolus-induced changes at early time points we first used linear interpolation to estimate the one hour value for base excess, chloride and bicarbonate based on the levels observed at baseline and 24 hours in the bolus and no bolus arms and a linear change in the levels from 1 to 24 hours:

<u>Imputation of base excess (be) at 1 hour</u>

be(1hr) = be(baseline) + slope + I(bolus) x (be(24hrs, bolus arm) - be(24hrs, no bolus arm))
where:
slope = (be(24hrs, no bolus arm) - be(baseline, no bolus arm)) / 24
I(bolus) = 1 for bolus sample and 0 otherwise

Chloride and bicarbonate were imputed similarly.
Haemoglobin was imputed using the values at 8 hours rather than 24 hours, in subjects who had not received blood transfusion.

Because the first approach is likely to underestimate the effects of albumin and saline on biochemical and acid-base status we also sought evidence from published studies to establish what changes should be expected at one hour after bolus. There is an extensive literature dating from the 1990s reporting hyperchloraemic acidosis occurring in recipients of normal saline or other high chloride containing solutions or comparing these solutions with buffered salt solutions (web extra Table 8). These studies include administration of crystalloids to healthy volunteers; administration of crystalloid solutions to patients undergoing a range of surgical conditions including both adults and children and ranging from gynaecological procedures, general surgical procedures, renal surgery and cardiac surgery; and studies in experimental animals. These studies establish that administration of normal saline and other high chloride containing fluids is invariably followed by hypochloraemic acidosis with a rise in chloride, decline in plasma bicarbonate and decrease in base excess occurring concurrently with the infusion and maximal immediately after the infusion. While the extensive literature has used a range of different infusion rates and volumes, those studies which have had comparable fluid volumes and rates to the 20-40 ml/kg infused during FEAST have shown decrease in base excess of approximately 5 mmol/l, a decline in bicarbonate of approximately 5 mmol/l and an increase in chloride of approximately 10 mmol/l in recipients of high chloride containing fluids. We therefore concluded there is strong evidence from the literature and our own data that the changes we have

detected in bolus recipients at the 24 hour time points are a minimum estimate of a predictable change induced by saline and albumin at earlier time points.
In order to provide a more realistic estimate based on the factors discussed above, we also estimated the 1 hour values for base excess, bicarbonate and chloride based on the reported changes after saline infusion in the many studies reported and summarised in web extra Table 8. We used the changes reported in those studies most similar to FEAST in the volume and timing of infusion.
Thus the estimates used in our analysis to impute the change in parameters from baseline to1 hour were as follows:

	**No bolus**	**Data estimate in bolus recipients**	**Literature estimate in bolus recipients**
**Base excess mEq/L**	+0·217	-1·025	-5
**Chloride mmol/L**	+0·011	+1·577	+10
**Bicarbonate mmol/L**	+0·194	-0·66	-5
**Haemoglobin g/L**	+0.03	-0.339	NA (-0.339*)

*The change in haemoglobin was derived only from data, and not from literature values, because measurements were available at 8 hours.

Cox proportional hazards regression modelling
To explore whether the changes induced by bolus at one hour could explain the adverse effect of bolus we built Cox proportional hazards survival models with the outcome as time of death. The baseline model contained only bolus as a covariate, covariates were added iteratively, and at each iteration the covariate with the smallest p-value for association with time of death in the multivariate survival model was added, until all covariates were added. In addition to a model using baseline variables, four sets of explanatory covariates representing the one hour time point were considered.

Covariate set 1
The first set of covariates contained the three scores at 1 hour and imputed levels for base excess, chloride, bicarbonate and haemoglobin based on the levels observed at baseline and 24 hours in the bolus and no bolus arms and a linear change in the levels from 1 to 24 hours.

Covariate set 2
In this set of covariates we used the scores and haemoglobin estimate as in covariate set 1 and imputed levels for base excess, chloride and bicarbonate in the bolus arm based on estimates of the effects of bolus on these blood parameters derived from published articles.

Estimates in the no bolus arm were the same as those used in covariate set 1.

Covariate set 3
The third set of covariates contained the individual components of the score and the imputed blood levels used in covariate set 1. The individual components of the score were respiratory rate above the norm for age, systolic blood pressure above and below the norm for age and heart rate above and below the norm for age.

Covariate set 4
Individual components of the score as used in covariate set 3 and the literature based estimates of the effects of bolus on the blood markers as used in covariate set 2.

The above models were refit including bolus as an additional explanatory covariate to calculate the hazard ratio for bolus under each model.

Web extra Figures

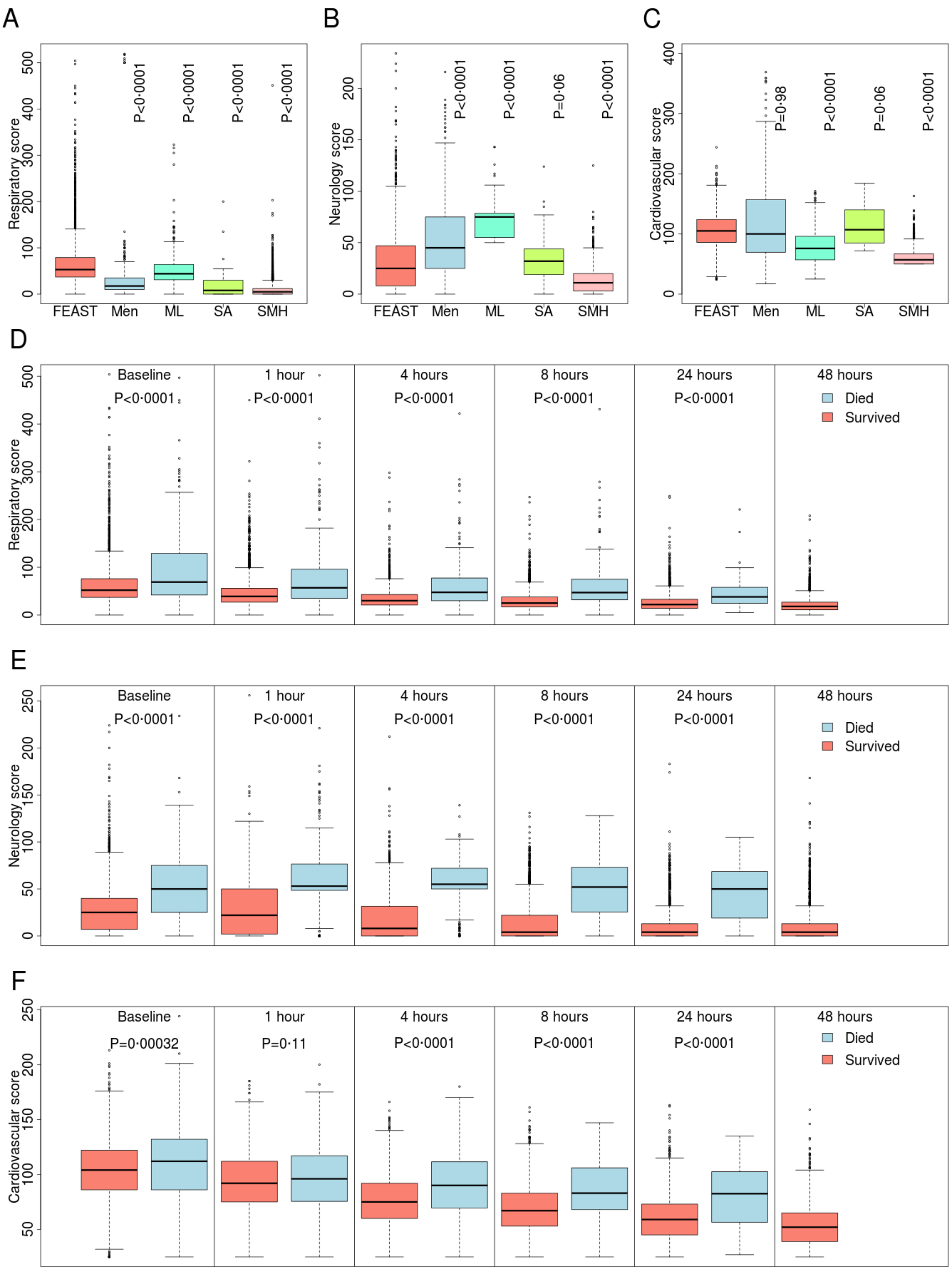

Web extra Figure 1. Physiological scores associated with aetiology and severity of illness across cohorts and sequential changes over time in FEAST

Respiratory (A), neurological (B) and cardiovascular (C) scores in FEAST and 4 other cohorts (Men, UK Meningococcal cohort; ML, Malawian cerebral malaria cohort; SA, South African sepsis cohort; SMH, St Mary's Hospital emergency department cohort). D-F show sequential changes in the FEAST cohort in respiratory (D), neurological score (E) and cardiovascular (F) scores at time points from admission baseline to 48 hours. Survivors are shown in red, cases dying in the next time period are shown in blue. Boxes show median and IQR; whiskers extend up to 1·5-times IQR. P for two-sided Mann-Witney test, unadjusted for multiple comparisons.

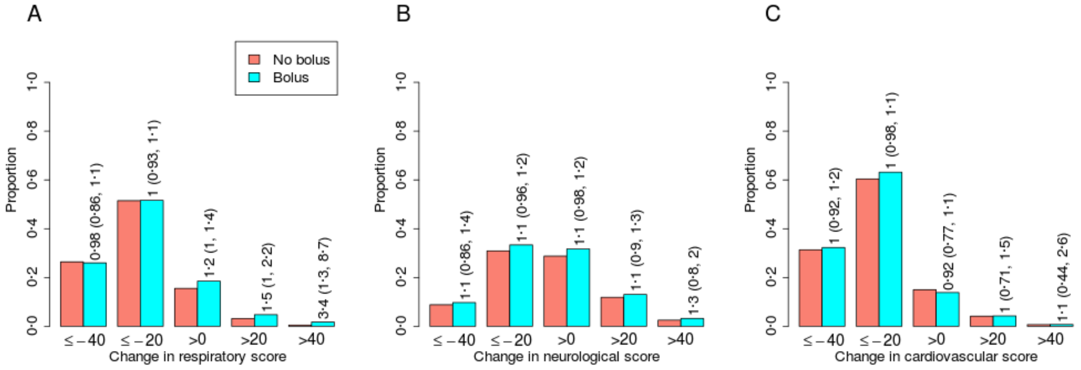

Web extra Figure 2. Change in physiological scores from baseline to 4 hours.
The proportion of individuals in FEAST with different magnitudes of change in physiological scores from baseline to four hours, compared between those randomized to no bolus (red bars) or bolus (blue bars). Negative values indicate decrease from the baseline, and positive values indicate increase from baseline. Values above bars show relative risk (95% CI) for comparison of proportions between bolus and no bolus groups.

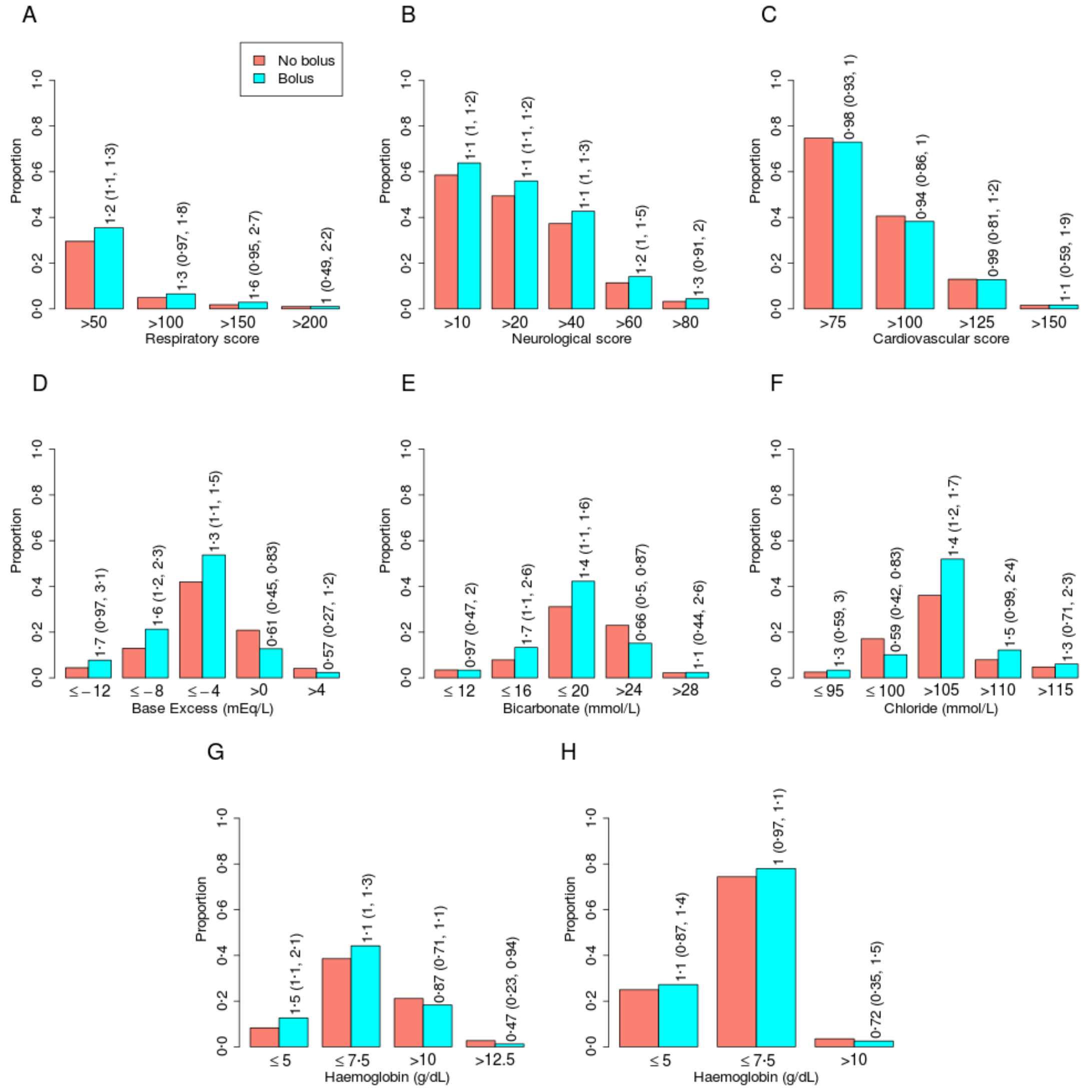

Web extra Figure 3. Distribution of physiological scores and blood parameters at first observation after randomization in FEAST.

Comparison of the proportion of individuals in FEAST with indicated values of physiological scores and blood measurements at the next observation after initiation of fluid bolus (blue) or no fluid bolus (red). A-C show physiological scores at 1 hour. D-F show biochemical measures at 24 hours. G and H show haemoglobin concentration at 8 hours in non-transfused (G) and transfused (H) subjects. Values above bars show relative risk (95% CI) for comparison of proportions between bolus and no bolus groups.

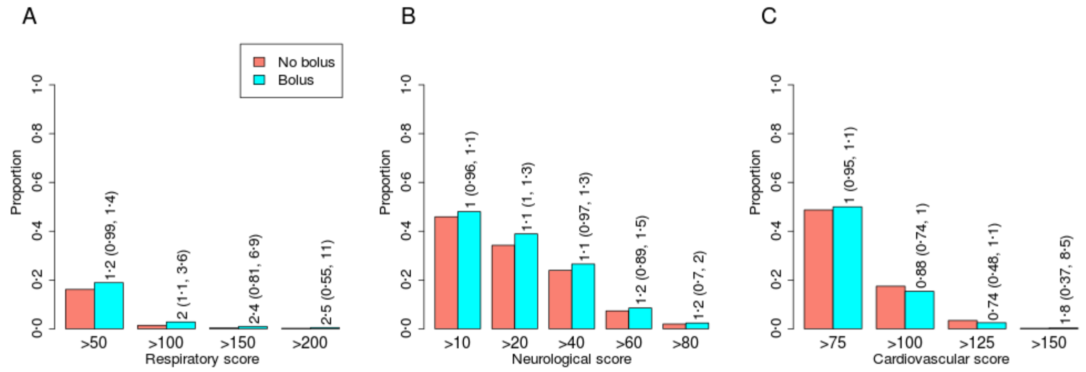

Web extra Figure 4. Distribution of physiological scores at 4 hours in FEAST.
Comparison of physiological scores at 4 hours after initiation of fluid bolus (blue) or no fluid bolus (red). Values above bars show relative risk (95% CI) for comparison of proportions between bolus and no bolus groups.

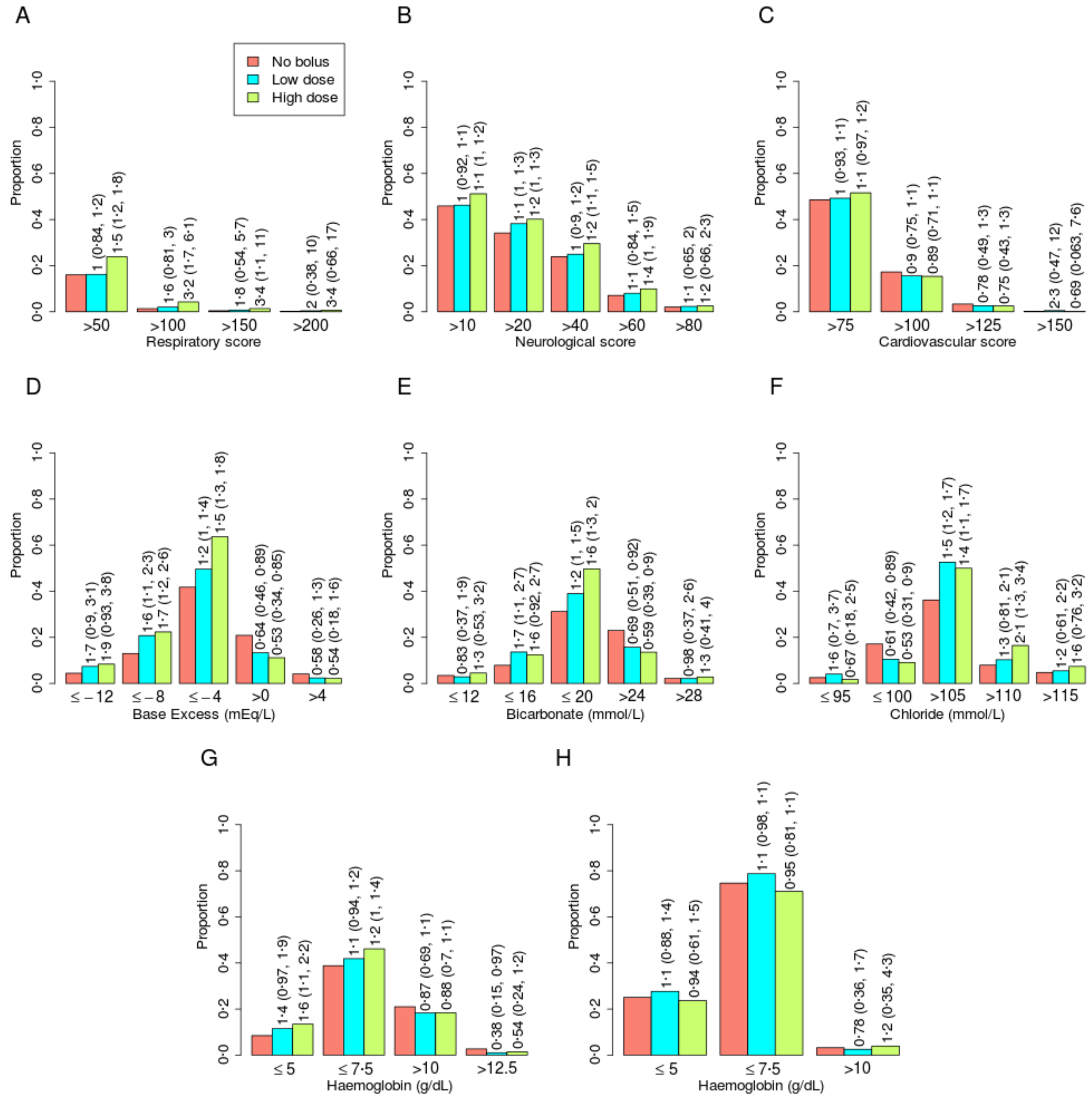

Web extra Figure 5. Distribution of physiological scores and blood parameters associated with volume of fluid bolus administered in FEAST.

The proportion of individuals in FEAST with indicated values of physiological scores and blood measurements at the next observation after completion of bolus fluids in all groups, in those who received high volume bolus (≥30ml/kg, green bars), low volume bolus (<30ml/kg, blue bars), or no bolus (red bars). A-C show physiological scores at 4 hours after bolus initiation. D-F show biochemical measures 24 hours after bolus initiation. G-H show haemoglobin concentration 8 hours after bolus initiation in non-transfused (G) and transfused (H) subjects. Values above bars show relative risks (95% CI) for comparison of proportions between low volume or high volume bolus groups vs no bolus groups.

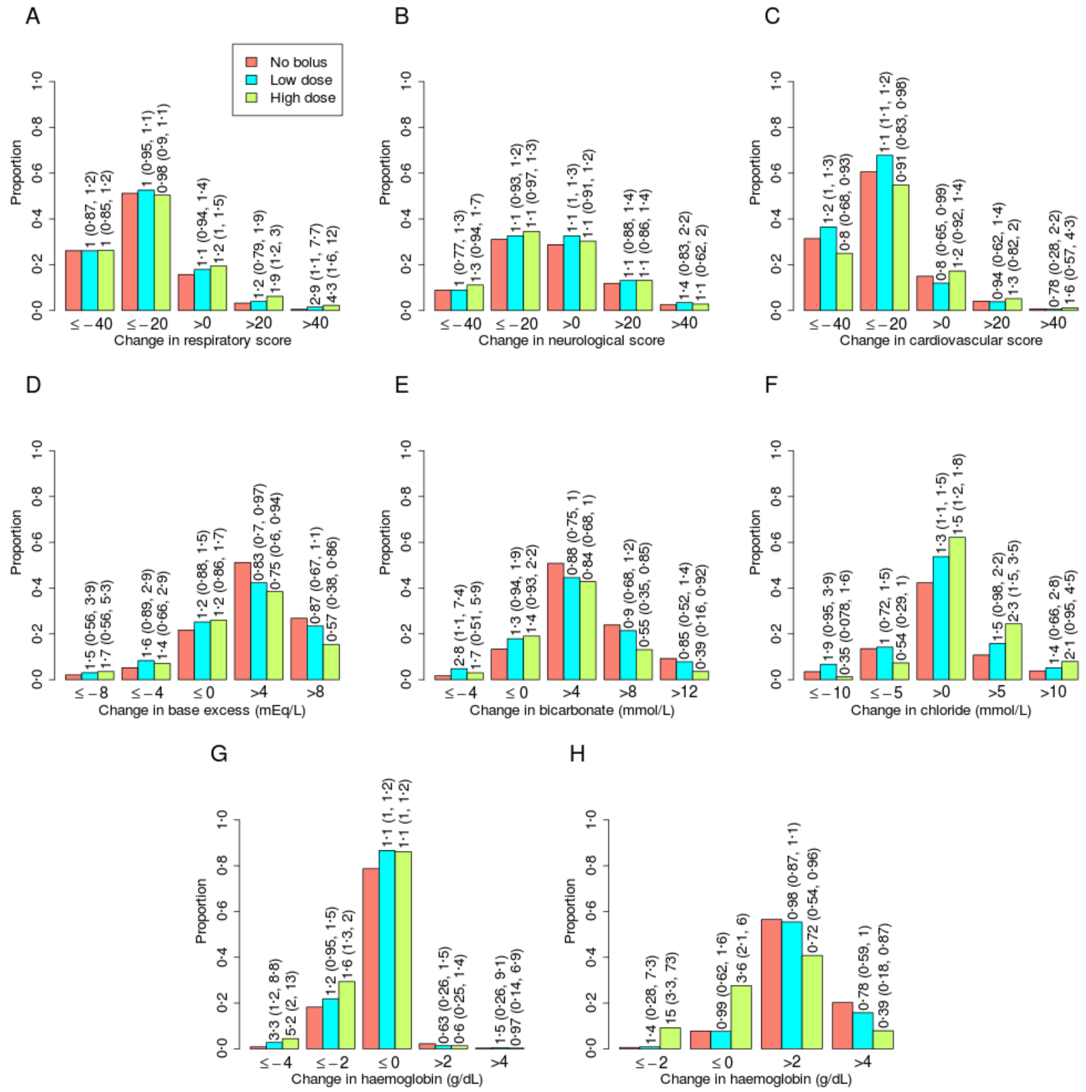

Web extra Figure 6. Changes from baseline to next observation for physiological scores and blood measures according to volume of fluid bolus received in FEAST.

Comparison of the proportion of individuals in FEAST with different magnitudes of change in physiological scores and blood measurements according to whether they received high volume bolus (≥30ml/kg, green bars), low volume bolus (<30ml/kg, blue bars) or no bolus (red bars). A-C show changes in physiological scores between 0 and 4 hours. D-F show changes in biochemical measures from baseline to 24 hours. G and H show change in haemoglobin from baseline to 8 hours in non-transfused (G) and transfused (H) subjects. The change from baseline is shown for each segment of the distribution. Negative values indicate decrease from the baseline, and positive values indicate increase from baseline. Values above bars show relative risks (95% CI) for comparison of proportions between low volume or high volume bolus groups vs. no bolus groups.

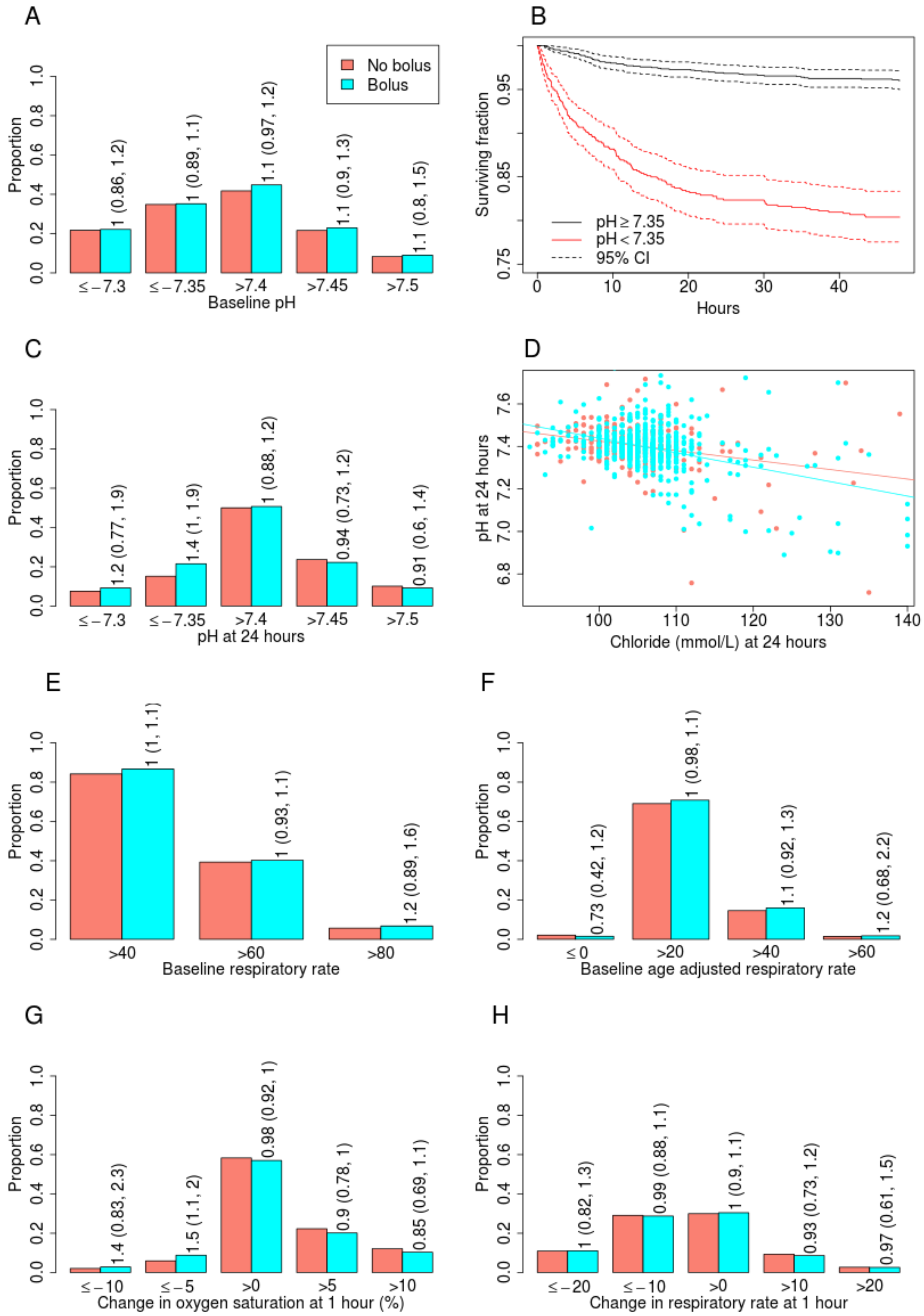

Web extra Figure 7. Hyperchloraemic acidosis in FEAST.
A shows distribution of pH at baseline in FEAST subjects according to whether they were randomized to no fluid bolus (red bars) or fluid bolus (blue bars). B shows survival in FEAST by baseline pH (black line, pH ≥7·35; red line pH <7·35; dotted lines 95% CI; Cox proportional hazards model with random effects for site; n=2082, 195 total events, p<0·0001). C shows proportion of individuals with indicated values of pH at 24 hours for no fluid bolus (red bars) or fluid bolus groups (blue bars). D shows correlation between chloride

concentration at 24 hours and pH at 24 hours shown by whether subjects were randomized to no fluid bolus (red dots and regression line, Pearson r=-0·28 (95% CI -0·38 to -0·17), p<0·0001) or fluid bolus (blue dots and regression line, Pearson r=-0·41 (95%CI -0·47 to -0·33), p<0.0001); p=0.05 for comparison of correlation coefficients using the z-test. E shows baseline respiratory rate and F the respiratory rate above mean for age. G-H show the proportion of individuals with different magnitudes of change from baseline to one hour in oxygen saturation (G) and respiratory rate (H) according to whether they were randomized to no fluid bolus (red bars) or fluid bolus (blue bars). Values above bars show relative risks (95% CI) for comparison of proportions between bolus vs no bolus groups.

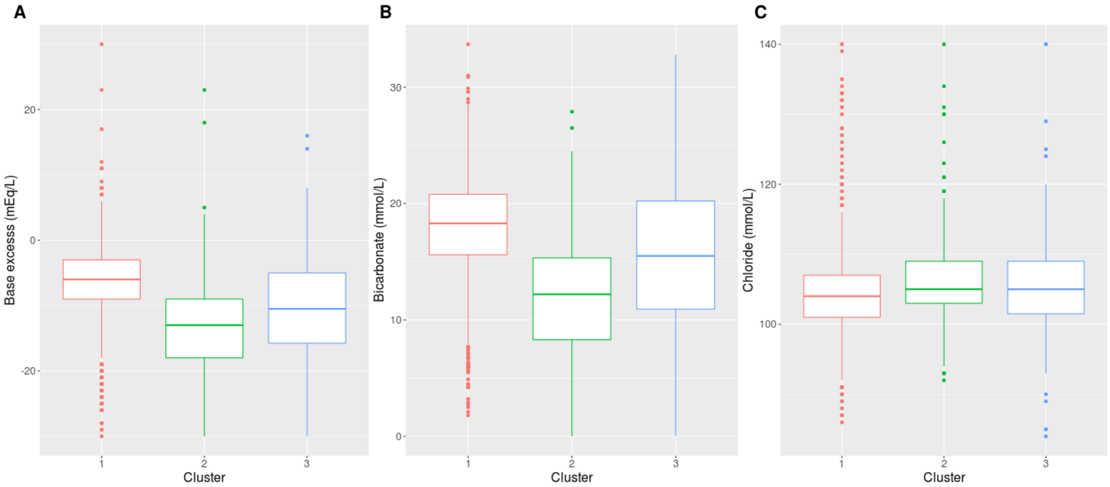

Web extra Figure 8. Biochemical measurements in clusters within FEAST.
Comparison of the distributions of baseline biochemical measurements by cluster within FEAST. Boxes show median and IQR; whiskers extend up to 1·5-times IQR.

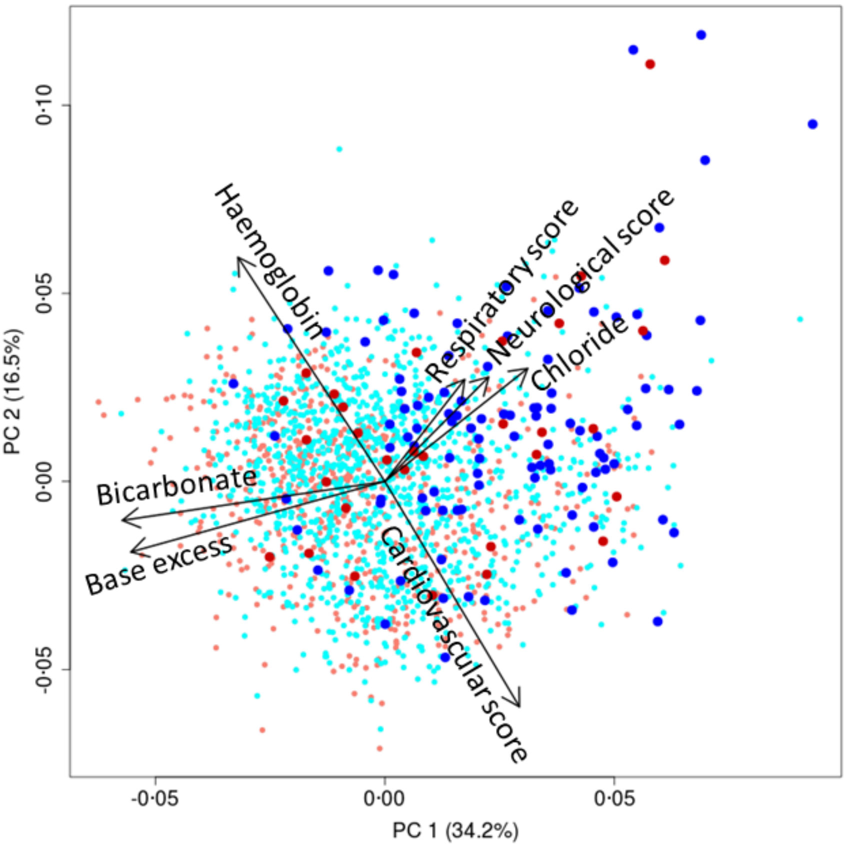

Web extra Figure 9. Principle component plot using physiological scores and blood parameters. Principal component (PC) analysis using the respiratory, neurological and cardiovascular scores at 1 hour after bolus, and conservative (data-derived) estimates of haemoglobin and biochemical values at 1 hour based on their values at 8 and 24 hours respectively (light red, no bolus, survivors; large dark red, no bolus, fatal cases; light blue, bolus, survivors; large dark blue, bolus, fatal cases). The principal component loadings (arrows) indicate the contributions of increasing values of each variable. Analysis is based on 1898 subjects with complete data for physiological scores at 1 hour and baseline biochemical parameters.

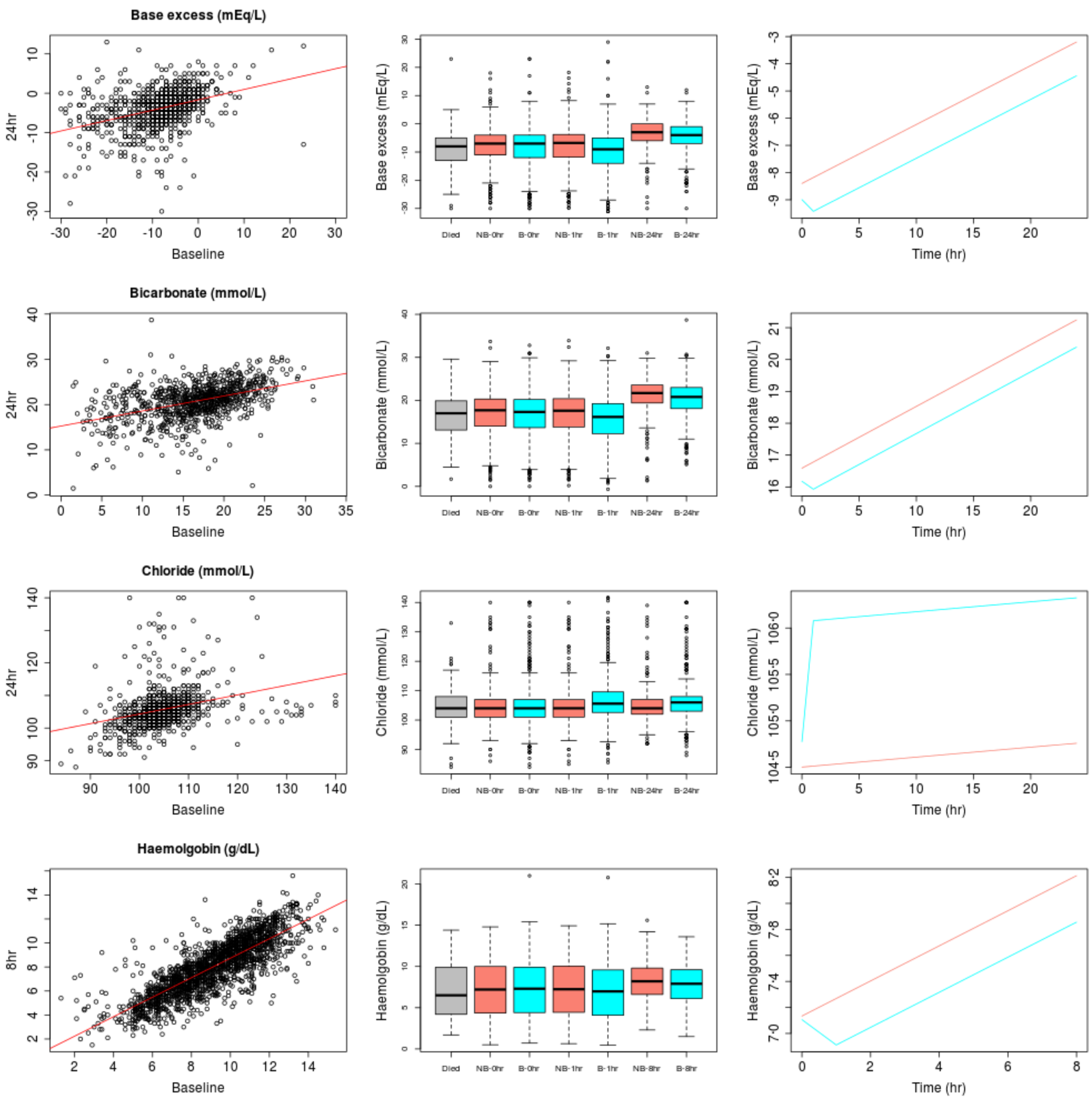

Web extra Figure 10. Imputation of blood parameters based on relationships between baseline and 24 hour values.

Relationships between baseline values and those at next measurement for base excess, chloride and bicarbonate (all 24 hours) and haemoglobin (8 hours) (first column of panels). Distribution of blood parameter values in subjects who died and those who survived at baseline, imputed from data at 1 hour, and at next measurement (second column of panels; NB (red), no bolus; B (blue), bolus; boxes show median and IQR; whiskers extend up to 1·5-times IQR). Illustration of imputation of values in bolus group based on difference in values between baseline and next measurement in no bolus group, and difference between bolus and no bolus groups at the second measurement timepoint (third column of panels).

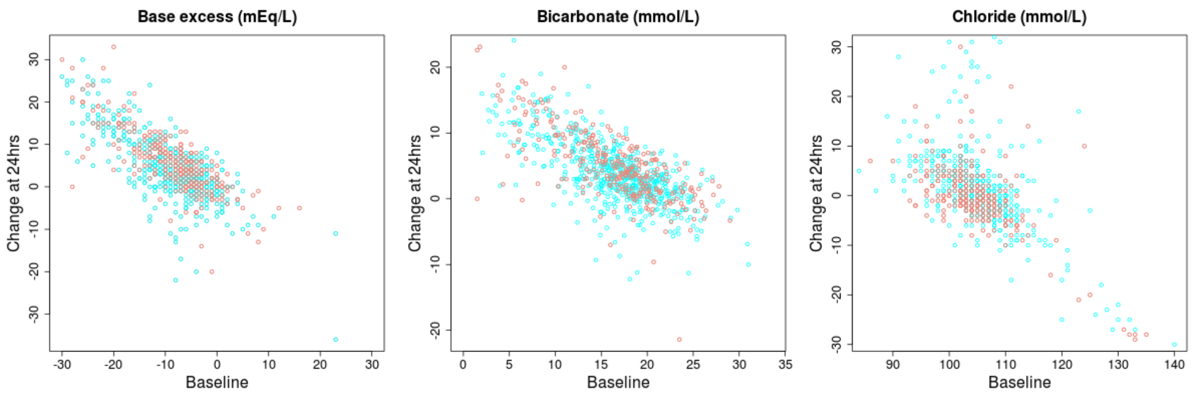

Web extra Figure 11. Relationship between baseline values and change at 24 hours for biochemical parameters.

Relationships between values at baseline and change from baseline to 24 hours for blood base excess, bicarbonate and chloride. Blue, bolus recipients; red no bolus.

Web extra Tables

Web extra Table 1. Numbers (%) of surviving subjects at each time point in FEAST Stratum A with available data for each variable.

	Baseline	1 hour	4 hours	8 hours	24 hours	48 hours
Surviving	3141	3102	3025	2974	2882	2844
Systolic blood pressure	3100 (99)	3037 (98)	2975 (98)	2923 (98)	2830 (98)	2772 (97)
Capillary refill time	3137 (100)	3078 (99)	3004 (99)	2950 (99)	2854 (99)	2787 (98)
Conscious level	3123 (99)	3069 (99)	2998 (99)	2943 (99)	2847 (99)	2776 (98)
Oxygen saturation	3038 (97)	3030 (98)	2979 (98)	2930 (99)	2834 (98)	2772 (97)
Heart rate	3141 (100)	3077 (99)	3002 (99)	2950 (99)	2853 (99)	2786 (98)
Respiratory rate	3124 (99)	3056 (99)	2997 (99)	2945 (99)	2845 (99)	2778 (98)
Respiratory score	3022 (96)	3008 (97)	2974 (98)	2925 (98)	2826 (98)	2763 (97)
Cardiovascular score	3096 (99)	3037 (98)	2975 (98)	2923 (98)	2829 (98)	2772 (97)
Neurological score	3082 (98)	3028 (98)	2969 (98)	2917 (98)	2822 (98)	2762 (97)
Hemoglobin	3054 (97)	0 (0)	0 (0)	2785 (94)	2744 (95)	0 (0)
Base excess	2070 (66)	0 (0)	0 (0)	0 (0)	914 (32)	0 (0)
Bicarbonate	2080 (66)	0 (0)	0 (0)	0 (0)	912 (32)	0 (0)
Chloride	2067 (66)	0 (0)	0 (0)	0 (0)	909 (32)	0 (0)

Web extra Table 2. Associations between physiological scores and outcome* in FEAST and 4 other cohorts.

Cohort	n (% all subjects)	Odds ratio for outcome per 10-unit increase in score	95% CI	P value
**Respiratory score**				
FEAST	3037 (96)	1·09	(1·07, 1·11)	<0·0001
Malawi	357 (80)	1·04	(0·99, 1·1)	0·13
South Africa	61 (100)	1·2	(0·97, 1·47)	0·089
Meningococcal	363 (72)	1·32	(1·19, 1·45)	<0·0001
SMH	13934 (74)	1·6	(1·54, 1.66)	<0·0001
**Neurology score**				
FEAST	3096 (98)	1·26	(1·21, 1·31)	<0·0001
Malawi	411 (92)	1·24	(1·08, 1·43)	0·0030
South Africa	61 (100)	1·6	(1·2, 2·14)	0·0010
Meningococcal	399 (79)	1·04	(0·99, 1·09)	0·16
SMH	2414 (13)	1·07	(0·97, 1·19)	0·16
**Cardiovascular score**				
FEAST	3110 (98)	1·09	(1·05, 1·14)	<0·0001
Malawi	411 (92)	1·19	(1·1, 1·28)	<0·0001
South Africa	61 (100)	1·04	(0·88, 1·22)	0·66
Meningococcal	333 (66)	1·08	(1·04, 1·12)	<0·0001
SMH	1509 (8)	1·3	(1·19, 1·42)	<0·0001

*Outcomes were defined as death in FEAST and the Meningococcal cohorts, death or neurological disability in the Malawian cohort, admission to intensive care in the South African cohort and admission to hospital or intensive care in the SMH emergency department cohorts. n, number of subjects with complete data available to calculate each score at baseline.

Web extra Table 3. Changes in physiological scores and blood parameters associated with albumin bolus or saline bolus in FEAST.

	Saline				Albumin				Albumin vs. saline
	n	Effect size	95% CI	p	n	Effect size	95% CI	p	p
**1 hour**									
Respiratory score	970	2·73	-0·23, 5·68	0·071	976	4·18	1·23, 7·13	0·0056	0·34
Neurological score	995	3·49	1·32, 5·67	0·0017	996	1·79	-0·39, 3·96	0·11	0·12
Cardiovascular score	1003	-2·34	-0·49, -4·20	0·014	1003	-2.0	-0·15, -3·86	0·035	0·72
**4 hours**									
Respiratory score	957	1·29	-1·02, 3·60	0·273	962	3·32	1·01, 5·63	0·0049	0·086
Neurological score	975	1·92	-0·088, 3·93	0·061	975	0·19	-1·82, 2·20	0·85	0·092
Cardiovascular score	982	-0·55	1·33, -2·44	0·56	981	-0·038	1·85, -1·92	0·97	0·59
**8 hours**									
Respiratory score	937	0·16	-2.00, 2·33	0·88	943	3.0	0·83, 5·16	0·0068	0·011
Neurological score	954	0·93	-0·94, 2·80	0·33	956	-0·29	1·58, -2·16	0·77	0·21
Cardiovascular score	960	-0·40	1·47, -2·28	0·67	962	0·61	-1·27, 2·48	0·53	0·29
Lactate mmol/L	895	-0.075	-0.32, 0.17	0.55	921	-0.16	-0.41, 0.081	0.19	0.48
Haemoglobin g/dL (untransfused)	949	-0.30	-0.45, -0.15	<0.0001	967	-0.49	-0.64, -0.34	<0.0001	0.012
Haemoglobin g/dL (transfused)	973	-0.34	-0.60, -0.079	0.0097	976	-0.12	-0.38, 0.14	0.37	0.095
**24 hours**									
Chloride mmol/L	265	2.1	1.15, 3.06	<0.0001	284	1.41	0.45, 2.37	0.0040	0.16
Bicarbonate mmol/L	270	-1.06	-1.65, -0.47	0.00057	294	-0.87	-1.46, -0.28	0.0039	0.53
Base excess mEq/L	271	-1.52	-2.27, -0.77	0.00010	294	-1.31	-2.05, -0.56	0.00065	0.579

Effect size is the mean change in the variable attributable to bolus. Positive effect size indicates increase in parameter, negative effect size indicates decrease, as compared with no bolus control.

Web extra Table 4. Association between changes in variables and risk of death in clusters within FEAST.

Group	n	OR	95% CI	p
**Respiratory score**				
FEAST	3037	1·09	1·07,1·11	<0·0001
Cluster 1	1967	1.00	0·90,1·11	0·98
Cluster 2	717	1·07	1,1·14	0·040
Cluster 3	353	1·03	1,1·06	0·020
**Neurology score**				
FEAST	3096	1·26	1·21,1·31	<0·0001
Cluster 1	1955	1·41	1·26,1·57	<0·0001
Cluster 2	772	1·30	1·2,1·41	<0·0001
Cluster 3	369	1·06	1·01,1·12	0·020
**Cardiovascular score**				
FEAST	3110	1·09	1·05,1·14	<0·0001
Cluster 1	1963	0·97	0·88,1·08	0·60
Cluster 2	777	1·05	0·98,1·12	0·16
Cluster 3	370	1·03	0·96,1·11	0·43
**Haemoglobin (g/dL)**				
FEAST	3082	0·88	0·85,0·91	<0·0001
Cluster 1	1929	1.00	0·91,1·1	0·97
Cluster 2	780	1·11	1·01,1·22	0·030
Cluster 3	373	0·91	0·85,0·98	0·008
**Lactate (mmol/L)**				
FEAST	3009	1·23	1·2,1·26	<0·0001
Cluster 1	1888	1·30	1·08,1·57	0·0060
Cluster 2	768	1·15	1·08,1·22	<0·0001
Cluster 3	353	1·15	1·09,1·22	<0·0001

OR, odds ratio for death for each ten-unit increase in the physiological scores and each unit increase in haemoglobin and lactate in FEAST overall and in clusters

Web extra Table 5. Changes in physiological scores and blood parameters associated with fluid bolus in FEAST Cluster 1.

Time (Hours)	n	Effect size	95% CI	p
**Respiratory score**				
1	1934	2·67	0·35, 4·99	0·020
4	1935	1·54	-0·50, 3·57	0·14
12	1916	0·87	-0·93, 2·67	0·34
**Neurology score**				
1	1926	3·42	1·38, 5·46	0·0010
4	1927	1·49	-0·47, 3·44	0· 13
12	1906	-0·14	-1·92, 1·64	0·87
**Cardiovascular score**				
1	1937	-1·72	-3·57, 0·14	0·070
4	1933	0·55	-1·37, 2·47	0·57
12	1915	0·66	1·24, 2·57	0·50
**Haemoglobin (g/dL)**				
8	1837	-0·35	-0·49-0·20	<0·0001
24	1818	-0·19	0·34, -0·035	0·020
**Lactate (mmol/L)**				
8	1806	-0·16	-0·39, 0·064	0· 15
24	1776	-0·070	-0·30, 0·16	0·50
**Base excess (mEql/L)**				
24	590	-1·34	-2·03, -0·64	0·0002
**Bicarbonate (mmol/L)**				
24	592	-0·90	-1·44, -0·36	0·0012
**Chloride (mmol/L)**				
24	575	1·99	0·46, 3·52	0·0010

*Effect size is the mean change in the variable attributable to bolus. Positive effect size indicates increase in parameter, negative effect size indicates decrease, as compared with no bolus control.

Web extra Table 6. Changes in physiological scores and blood parameters associated with fluid bolus in FEAST Cluster 2.

Time (Hours)	n	Effect size	95% CI	p
**Respiratory score**				
1	676	5·11	-0·37, 10·6	0·06
4	655	3·8	0·038, 7·6	0·040
12	634	1·1	-2·79, 5·02	0·50
**Neurology score**				
1	730	4·32	0·41, 8·22	0·030
4	698	1·19	-2·48, 4·86	0· 15
12	676	0·57	-3·01, 4·16	0·70
**Cardiac score**				
1	737	-1·75	-5·24, 1·75	0·32
4	706	-0·65	-4·08, 2·78	0·71
12	683	-0·33	-3·87, 3·21	0·85
**Haemoglobin (g/dL)**				
8	646	-0·16	-0·45, 0·13	0·27
24	628	-0·091	-0·38, 0·2	0·54
**Lactate (mmol/L)**				
8	654	-0·041	-0·58, 0·50	0· 88
24	616	0·030	-0·42, 0·48	0·90
**Base excess (mEql/L)**				
24	182	-1·34	-3·46, -0·021	0·050
**Bicarbonate (mmol/L)**				
24	180	-1·74	-2·62, 0·082	0·070
**Chloride (mmol/L)**				
24	180	2·87	0·73, 5·02	0·0090

*Effect size is the mean change in the variable attributable to bolus. Positive effect size indicates increase in parameter, negative effect size indicates decrease, as compared with no bolus control.

Web extra Table 7. Changes in physiological scores and blood parameters associated with fluid bolus in FEAST Cluster 3.

Time (Hours)	n	Effect size	95% CI	p
**Respiratory score**				
1	315	3·44	-10·9, 17·8	0· 63
4	294	3·69	-7·65, 15	0· 52
12	288	7·13	-3·86, 18·1	0·20
**Neurology score**				
1	336	-5·19	-12·9, 2·55	0·19
4	308	-1·32	-8·46, 5·83	0· 71
12	299	2·98	-3·79, 9·76	0·38
**Cardiac score**				
1	336	-4·84	-10·2, 0·56	0·08
4	309	-4·23	-10·1, 1·6	0·15
12	299	-2·06	-7·8, 3·68	0·48
**Haemoglobin (g/dL)**				
8	288	-0·52	-0·92, -0·12	0·010
24	277	-0·59	-1·02, -0·16	0·008
**Lactate (mmol/L)**				
8	277	0·0022	-0·75, 0·76	0· 99
24	265	0·30	-0·40, 1.00	0·40
**Base excess (mEql/L)**				
24	85	-1·09	-3·68, 1·5	0·41
**Bicarbonate (mmol/L)**				
24	86	-0·69	-2·69, 1·32	0·50
**Chloride (mmol/L)**				
24	85	-3·04	-5·94, -0·15	0·040

*Effect size is the mean change in the variable attributable to bolus. Positive effect size indicates increase in parameter, negative effect size indicates decrease, as compared with no bolus control.

Web extra Table 8. Studies evaluating the effects of unbalanced salt solutions on blood acid-base balance and chloride

Paper	Author	Year	Fluid volume	Study Type/Detail	Bicarbonate	Base Excess	Chloride
**Balanced Crystalloids Versus Saline for Perioperative Intravenous Fluid Administration in Children Undergoing Neurosurgery: A Randomized Clinical Trial[23]**	Lima et al.	2019	"4-2-1 rule"	RCT: 53 paediatric patients having neurosurgery randomised to saline or balanced crystalloid.		post-preop change in BE: −4.4 [IQR −5.0; −2.3] in saline group vs. −0.4 [−2.7; 1.3] mmol/L in balanced group; P < 0.001	post-preop change in chloride: 6 [IQR 3.5; 8.5] mmol/L in saline group compared with 0 [−1.0; 3.0] mmol/L in balanced group; P < 0.001
**Balanced Crystalloids versus Saline in Critically Ill Adults[24]**	Semler et al.	2018	Median volume saline given 1020mls	Cluster-randomized, multiple-crossover trial of 15,802 adult ICU patients. Randomised to saline or balanced crystalloid.	Lowest level between enrolment and day 30, median [IQR]: Balanced group 21.0 mmol/L [18.0 – 23.0] vs 20.0 in saline group [17.0 – 22.0]. p <0.001<br><br>Bicarb < 20 mmol/L between enrolment and day 30, No. (%): balanced group 2793 (35.2) vs 3307 (42.1) in saline group. p<0.001		Highest level between enrolment and day 30, median [IQR]. Balanced group 108 mmol/L [105 – 111] vs saline group 109 [105 – 112]. P<0.001<br><br>Cl >110 mmol/L between enrolment and day 30, No. (%) 1945 (24.5) in balanced group vs 2796 (35.6) in saline group. P<0.001
**Balanced Crystalloids versus Saline in Noncritically Ill Adults[25]**	Self et al.	2018	Median volume in ED 1079 mls	Multiple crossover trial of 13,347 adults requiring hospital but not ICU admission	Lowest bicarb <20mmol/L: 1668 (24.9%) in balanced group vs 1859 (28%) in saline group. P<0.01		Highest chloride >110 mmol/L: 1020 (15.2%) in balanced group vs 1280 (19.3%) in saline group. P<0.01
**Normal saline versus a balanced crystalloid for goal-directed perioperative fluid therapy in major abdominal surgery: a double-blind randomized[26]**	Pfortmueller et al.	2018	2 ml/kg ideal body weight/hr (increased if viscera exposed)	RCT of adults undergoing major abdominal surgery		Median minimum base excess was lower in the saline group than the balanced group: -6.0 (-12 to 4) vs 0.0 (-5 to 3) mmol/L P<0.0001	Intra-operative change in chloride 7 mmol/L (2-16) in saline group compared to 2mmol/L (0-9) in the balanced group p<0.0001
**A randomized trial of Plasma-Lyte A and 0.9 % sodium chloride in acute paediatric gastroenteritis[27]**	Allen et al.	2016	No standard regime	RCT of children with moderate to severe dehydration.	Mean change in bicarb 1.6 mEq/L in balanced group vs 0mEq/L in saline group (p=0.004)		Balanced group: baseline Cl 103.03 mmol/L +/- 4.74 to 104.49 +/- 3.18 at 4 hours. Saline group 103.53 +/- 4.19 to 108.51 +/- 4.87. P<0.001

**Effects of Fluid Resuscitation With 0.9% Saline Versus a Balanced Electrolyte Solution on Acute Kidney Injury in a Rat Model of Sepsis[28]**	Zhou et al.	2014	10 mL/kg in the first hour and 5 mL/kg in the next 3 hr	Controlled laboratory experiment of 60 adult rats		In saline group, BE changed following resuscitation (2 vs 5 mmol/L; $p < 0.05$)	Saline group: chloride increased (109 vs 102 mmol/L; $p < 0.05$), Balanced fluid group: no hyperchloremia (102 vs 101 mmol/L; $p > 0.05$)
**Saline Versus Plasma-Lyte A in Initial Resuscitation of Trauma Patients[29]**	Young et al.	2014	No standard regime	RCT of adult trauma patients. Plasmalyte vs 0.9% saline	Change in bicarbonate in saline group at 24 hours 22 ± 4 mEgl/L compared to 26 ± 3 in balanced group.	Change in BE in saline group at 24 hours 4.4 ± 3.9 mmol/L compared to 7.5 ± 4.7 in balanced group	At 24 hours saline group 111 +/- 8 mEq/L vs 104 +/- 4 in plasma-lyte group. Difference of -7 (95% CI -10 - -3).
**A novel balanced isotonic sodium solution vs normal saline during major surgery in children up to 36 months: a multicentre RCT[30]**	Disma et al.	2014	4 ml/kg/h for the first 10 kg, 2 ml/kg/h from 11 to 20 kg and 1 ml/kg/h for every kg more than 20 kg	RCT: 240 paediatric patients undergoing major surgery. Balanced crystalloid plus 1% glucose vs saline + 1% glucose		Median change balanced solution -0.95 vs -1.7 in saline group (p=0.019)	Median change 4 mEq/L in the saline group compared to 2 in balanced group (p= 0.0001)
**Comparisons of normal saline and lactated Ringer's resuscitation on hemodynamics, metabolic responses, and coagulation in pigs after severe hemorrhagic shock.[31]**	Martini et al.	2013	To match MAP achieved in a pig resus with 3*bled volume of RL	Randomised trial of 20 pigs. Induced haemorrhage following by resuscitation with Ringer's lactate (RL) or normal saline (NS)	Bicabronate drop in RL and NS group. Bicarbonate remained lower in NS group compared to RL group at 3 and 6 hours (P<0.05)	Base excess drop in RL and NS group and remained lower in NS group compared to RL group at 3 and 6 hours (P<0.05)	Hyperchloraemia for 6 h after NS resuscitation (102 mM +/-3 at baseline to 123 +/- 3 at 6 hours),. Not seen after RL resuscitation (101 mM +/- 2 at baseline to 97 +/-3 at 6 hrs).
**Balanced versus chloride-rich solutions for fluid resuscitation in brain-injured patients: a randomised double-blind pilot study.[32]**	Roquilly et al.	2013	30ml/kg/day	RCT: 42 patients with severe traumatic brain injury randomised to isotonic balanced solutions or isotonic sodium chloride solutions		Saline group: Median (IQR) BE at baseline -1.7 (-3.9 - -0.3) to -2.4 (-3.7 - -0.9) at 6 hrs vs balanced group -2.6 (-3.5 - -0.1) to -0.3 (-1.3 - 1) at 6 hrs (p=0.004)	Higher incidence of hyperchloremic acidosis in the saline than balanced fluid group ($P = 0.01$). Mean difference in chloride between the saline and balanced group of 4.8 mmol/L (1.9 to 7.6); $P = 0.002$
**A Randomized, Controlled, Double-Blind Crossover Study on the Effects of 2L Infusions of 0.9% Saline and Plasma-Lyte 148 on Renal Blood Flow Velocity and Renal**	Chowdhury et al.	2012	2L over 1 hour	12 healthy adult males			Chloride peaked at 109mol/L in saline group. Remained high for duration of study. Levels remained normal in plasmalyte group (p<0.0001)

**Cortical Tissue Perfusion in Healthy Volunteers.**[33]							
**The biochemical effects of restricting chloride-rich fluids in intensive care.**[34]	Yunos et al.	2011	No standard fluid prescriptio n	Prospective, open-label, before-and-after study. Significant reduction in use of chloride rich fluids between control (828 patients) and intervention (816 patients) periods.	Time weighted mean (SD) pre-intervention 25.3 mmol/L (4.0) and 26.4 (4.1) after (P<0.001).	Pre-intervention (no limitation on chloride rich fluids) 9.1% had a base excess <-5mEq/L compared to 6% following intervention (P<0.001). Time weighted mean (SD) pre-intervention 0.5 mmol/L (4.5) and 1.8 (4.7) after (P<0.001).	Severe hyperchloraemia (>114mmol/L) reduced from 6.2% pre-intervention to 2.3% following intervention (P<0.001). Time weighted mean (SD) pre-intervention 104.9 mmol/L (4.9) and 102.5 (4.6) after (P<0.001).
**Resuscitation with balanced electrolyte solution prevents hyperchloremic metabolic acidosis in patients with diabetic ketoacidosis.**[35]	Mahler et al.	2010	20ml/kg bolus then DKA protocol rates	RCT of the resuscitation of adults with DKA. Normal saline vs plasma-lyte A	Mean post-resuscitation bicarb 17 mmol/L (95% CI 15-18) in saline group vs 20 (18-21) in plasmalyte group. P=0.02		Mean post-resuscitation chloride 111 mmol/L (95% CI 110-112) in saline group vs 105 (103-108) in plasmalyte group. P<0.001
**Effect of volume loading with 1 liter intravenous infusions of 0.9% saline, 4% succinylated gelatine (Gelofusine) and 6% hydroxyethyl starch (Voluven) on blood volume and endocrine responses: A randomized, three-way crossover study in healthy volunteers.**[36]	Lobo et al.	2010	1L over 1 hr	Randomized, three-way crossover study of 10 healthy adult males.	Reduction in bicarbonate after infusion but non-significant chance between saline and voluven or gelofusin.		Persistent hyperchloremia more marked after 0.9% saline and Voluven than Gelofusine. Saline vs gelofusin p=0.08
**Lactated Ringer's is Superior to Normal Saline in the Resuscitation of Uncontrolled Hemorrhagic Shock.**[37]	Todd et al.	2007	Fluids to target MBP 256.3 +/- 145.4 mL/kg of fluid	Randomised study of 20 adult swine		Final BE in saline group -4.6 +/- 7.9 mmol/L compared to 7.2 +/- 4.2 in RL group (p<0.01)	Final chloride in saline group 119 +/- 5.6 mEq/L compared to 105 +/- 2.9 in RL group (p<0.0001)
**(Ab)normal saline and physiological Hartmann's solution: a randomized double-blind crossover study.**[38]	Reid et al.	2003	2L infusion over an hours	Double-blind crossover study of 9 healthy adult males	Saline group: Mean 26.7 mmol/L to 25.6 at one hour; Hartmann's group 26.9 to 27.1 mmol/L (p=0.008)		Saline group: Mean 103 mmol/L to 108 at one hour; Hartmann's group 103 to 104 mmol/L (p<0.001)
**The Effects of Balanced Versus Saline-Based Hetastarch and Crystalloid Solutions on**	Wilkes et al.	2001	500ml bolus then 7ml/kg/hr	Randomised trial of 47 adult patients.	Pre to post op change in balanced group: 25.8 +/- 3.3 to 24.7 +/- 3. Pre to post op change in saline	Pre to post op change in balanced group: 0.7 +/- 2.7 to -0.2 +/- 2.6. Pre to post op change in	Pre to post op change in balanced group: 104.9 +/- 3.2 to 108.2 +/1 3.4. Pre to post op change in

**Acid-Base and Electrolyte Status and Gastric Mucosal Perfusion in Elderly Surgical Patients.**[39]					group: 25.7 +/- 2.6 to 21.8 +/- 3.2 (p<0.0073)	saline group: 1.7 +/- 2.3 to -3.8 +/- 2.9 (p=0.0001)	saline group: 104.2 +/- 3.5 to 114 +/- 4.9 (p=0.0001)
**Rapid Saline Infusion Produces Hyperchloremic Acidosis in Patients Undergoing Gynecologic Surgery.**[40]	Scheingraber et al.	1999	30ml/kg/hr	Randomised trial: 24 women received saline or ringer's lactate (RL). Comparison between 0 and 120 mins	Calculated using Henderson—Hasselbach. In saline group change from 23.5 mM +/- 2.2 to 18.4 +/- 2. In RL group 23.3 +/- 2.0 to 23.0 +/- 1.1	Mean change of -6.3 mM in saline group. 'No major change' in RL group.	Change from a mean of 104 to-115 mM in saline group and a mean of 104 – 106 mM in RL group.
**A comparison of Plasmalyte 148 and 0.9% saline for intra-operative fluid replacement.**[41]	McFarlane et al.	1994	15ml/kg/hr during surgery	Randomised trial of 30 patients undergoing major surgery.	Difference between mean pre and post op values in saline group: -4 mmol/L +/- 2.0; Plasmalyte group -0.7 +/- 1.0 (p<0.01)	Difference between mean pre and post op values in saline group: -5 +/- 2.1; Plasmalyte group -1.2 +/- 1.1 (p<0.01)	Difference between mean pre and post op values in saline group: 6.9 mmol/L +/- 2.03; Plasmalyte group 0.6 +/- 1.2 (p<0.01)

RCT, Randomised controlled trial; RL, Ringer's lactate; op, operative. Blank cells indicate that the parameter was not assessed.

Web extra Table 9. Cox proportional hazard regression models for time of death.

Covariate	Coefficient	p
Model using values at baseline with composite physiological scores		
Bolus – literature derived estimates	0·50	0·011
Neurological score	0·015	<0·0001
Respiratory score	0·0019	0·075
Base excess	-0·024	0·34
Bicarbonate	-0·097	0·0065
Chloride	0·019	0·095
Haemoglobin	-0·015	0·62
Cardiovascular score	0·00093	0·77
Model using values at 1 hour with composite physiological scores (covariate sets 1 & 2)		
Bolus – data derived estimates	0·17	0·42
Bolus – literature derived estimates	-0·37	0·15
Neurological score	0·026	<0·0001
Respiratory score	0·0082	<0·0001
Base excess	-0·029	0·25
Bicarbonate	-0·067	0·068
Chloride	0·016	0·25
Haemoglobin	0·020	0·50
Cardiovascular score	-0·00086	0·79
Model using values at 1 hour with component physiological variables (covariate sets 3 & 4)		
Bolus – data derived estimates	0·22	0·30
Bolus – literature derived estimates	-0·33	0·20
Heart rate - down	0·039	<0·0001
Base excess	-0·027	0·28
AVPU	0·75	<0·0001
O_2 saturation	-0·041	<0·0001
SBP - up	0·021	<0·0001
Bicarbonate	-0·066	0·066
SBP - down	0·026	0·092
Chloride	0·018	0·18
Respiratory rate - up	0·0066	0·25
Haemoglobin	0·034	0·27
Heart rate - up	-0·0025	0·59
Capillary refill time	0·019	0·88

Cox-proportional hazards regression coefficients and p-values for association with time of death in a multivariate model with all listed covariates included. Coefficients and p-values for all covariates in sets1 and 2, except bolus, are identical. Similarly for covariate sets 3 and 4.

References

1. Maitland K, Kiguli S, Opoka RO, et al. Mortality after fluid bolus in african children with severe infection. *N Engl J Med* 2011; **364**(26): 2483-95.
2. Fleming S, Thompson M, Stevens R, et al. Normal ranges of heart rate and respiratory rate in children from birth to 18 years of age: A systematic review of observational studies. *Lancet* 2011; **377**(9770): 1011-8.
3. National High Blood Pressure Education Program Working Group on High Blood Pressure in Children and Adolescents. The fourth report on the diagnosis, evaluation, and treatment of high blood pressure in children and adolescents. *Pediatrics* 2004; **114**(2 Suppl 4th Report): 555-76.
4. Cecconi M, De Backer D, Antonelli M, et al. Consensus on circulatory shock and hemodynamic monitoring. Task force of the European Society of Intensive Care Medicine. *Intensive Care Med* 2014; **40**(12): 1795-815.
5. Davis AL, Carcillo JA, Aneja RK, et al. American College of Critical Care Medicine clinical practice parameters for hemodynamic support of pediatric and neonatal septic shock. *Crit Care Med* 2017; **45**(6): 1061-93.
6. Maitland K, George EC, Evans JA, et al. Exploring mechanisms of excess mortality with early fluid resuscitation: Insights from the feast trial. *BMC Med* 2013; **11**: 68.
7. Houston KA, George EC, Maitland K. Implications for paediatric shock management in resource-limited settings: A perspective from the feast trial. *Crit Care* 2018; **22**(1): 119.
8. Advanced Life Support Group Advanced paediatric life support: A practical approach to emergencies. 6th edition ed: Wiley-Blackwell; 2016.
9. Kissoon N, Orr RA, Carcillo JA. Updated american college of critical care medicine--pediatric advanced life support guidelines for management of pediatric and neonatal septic shock: Relevance to the emergency care clinician. *Pediatr Emerg Care* 2010; **26**(11): 867-9.
10. World Health Organization. Pocket book of hospital care for children: Guidelines for the management of common childhood illnesses. WHO 2013.
11. Goldstein B, Giroir B, Randolph A, International Consensus Conference on Pediatric S. International pediatric sepsis consensus conference: Definitions for sepsis and organ dysfunction in pediatrics. *Pediatr Crit Care Med* 2005; **6**(1): 2-8.
12. Singer M, Deutschman CS, Seymour CW, et al. The third international consensus definitions for sepsis and septic shock (sepsis-3). *JAMA* 2016; **315**(8): 801-10.
13. Levy MM, Fink MP, Marshall JC, et al. 2001 sccm/esicm/accp/ats/sis international sepsis definitions conference. *Intensive Care Med* 2003; **29**(4): 530-8.
14. Burri C, Ladrach HR, Siegrist J, Allgower M. [significance of the arterial blood pressure, its amplitude and pulse rate in the hypovolemic patient]. *Helv Chir Acta* 1967; **34**(6): 535-50.
15. Acker SN, Bredbeck B, Partrick DA, Kulungowski AM, Barnett CC, Bensard DD. Shock index, pediatric age-adjusted (SIPA) is more accurate than age-adjusted hypotension for trauma team activation. *Surgery* 2017; **161**(3): 803-7.
16. Robba C, Bacigaluppi S, Cardim D, Donnelly J, Bertuccio A, Czosnyka M. Non-invasive assessment of intracranial pressure. *Acta Neurol Scand* 2016; **134**(1): 4-21.
17. Kalmar AF, Van Aken J, Caemaert J, Mortier EP, Struys MM. Value of Cushing reflex as warning sign for brain ischaemia during neuroendoscopy. *Br J Anaesth* 2005; **94**(6): 791-9.
18. Yumoto T, Mitsuhashi T, Yamakawa Y, et al. Impact of Cushing's sign in the prehospital setting on predicting the need for immediate neurosurgical intervention in trauma patients: A nationwide retrospective observational study. *Scand J Trauma Resusc Emerg Med* 2016; **24**(1): 147.
19. Su CF, Yang YL, Lee MC, Chen HI. A severe vicious cycle in uncontrolled subarachnoid hemorrhage: The effects on cerebral blood flow and hemodynamic responses upon intracranial hypertension. *Chin J Physiol* 2006; **49**(1): 56-63.
20. Molyneux E, Walsh A, Phiri A, Molyneux M. Acute bacterial meningitis in children admitted to the Queen Elizabeth Central Hospital, Blantyre, Malawi in 1996-97. *Trop Med Int Health* 1998; **3**(8): 610-8.
21. Antoniak CE. Mixtures of dirichlet processes with applications to bayesian nonparametric problems. *Annals of Statistics* 1974; **2**(6): 1152-74.
22. Liverani S, Hastie DI, Azizi L, Papathomas M, Richardson S. Premium: An R package for profile regression mixture models using dirichlet processes. *J Stat Softw* 2015; **64**(7): 1-30.
23. Lima MF, Neville IS, Cavalheiro S, Bourguignon DC, Pelosi P, Malbouisson LMS. Balanced crystalloids versus saline for perioperative intravenous fluid administration in children undergoing neurosurgery: A randomized clinical trial. *J Neurosurg Anesthesiol* 2019; **31**(1): 30-5.

24. Semler MW, Self WH, Wanderer JP, et al. Balanced crystalloids versus saline in critically ill adults. *N Engl J Med* 2018; **378**(9): 829-39.
25. Self WH, Semler MW, Wanderer JP, et al. Balanced crystalloids versus saline in noncritically ill adults. *N Engl J Med* 2018; **378**(9): 819-28.
26. Pfortmueller CA, Funk GC, Reiterer C, et al. Normal saline versus a balanced crystalloid for goal-directed perioperative fluid therapy in major abdominal surgery: A double-blind randomised controlled study. *Br J Anaesth* 2018; **120**(2): 274-83.
27. Allen CH, Goldman RD, Bhatt S, et al. A randomized trial of plasma-lyte a and 0.9 % sodium chloride in acute pediatric gastroenteritis. *BMC Pediatr* 2016; **16**: 117.
28. Zhou F, Peng ZY, Bishop JV, Cove ME, Singbartl K, Kellum JA. Effects of fluid resuscitation with 0.9% saline versus a balanced electrolyte solution on acute kidney injury in a rat model of sepsis. *Crit Care Med* 2014; **42**(4): e270-8.
29. Young JB, Utter GH, Schermer CR, et al. Saline versus plasma-lyte a in initial resuscitation of trauma patients: A randomized trial. *Ann Surg* 2014; **259**(2): 255-62.
30. Disma N, Mameli L, Pistorio A, et al. A novel balanced isotonic sodium solution vs normal saline during major surgery in children up to 36 months: A multicenter RCT. *Paediatr Anaesth* 2014; **24**(9): 980-6.
31. Martini WZ, Cortez DS, Dubick MA. Comparisons of normal saline and lactated Ringer's resuscitation on hemodynamics, metabolic responses, and coagulation in pigs after severe hemorrhagic shock. *Scand J Trauma Resusc Emerg Med* 2013; **21**: 86.
32. Roquilly A, Loutrel O, Cinotti R, et al. Balanced versus chloride-rich solutions for fluid resuscitation in brain-injured patients: A randomised double-blind pilot study. *Crit Care* 2013; **17**(2): R77.
33. Chowdhury AH, Cox EF, Francis ST, Lobo DN. A randomized, controlled, double-blind crossover study on the effects of 2-l infusions of 0.9% saline and Plasma-lyte(r) 148 on renal blood flow velocity and renal cortical tissue perfusion in healthy volunteers. *Ann Surg* 2012; **256**(1): 18-24.
34. Yunos NM, Kim IB, Bellomo R, et al. The biochemical effects of restricting chloride-rich fluids in intensive care. *Crit Care Med* 2011; **39**(11): 2419-24.
35. Mahler SA, Conrad SA, Wang H, Arnold TC. Resuscitation with balanced electrolyte solution prevents hyperchloremic metabolic acidosis in patients with diabetic ketoacidosis. *Am J Emerg Med* 2011; **29**(6): 670-4.
36. Lobo DN, Stanga Z, Aloysius MM, et al. Effect of volume loading with 1 liter intravenous infusions of 0.9% saline, 4% succinylated gelatine (gelofusine) and 6% hydroxyethyl starch (voluven) on blood volume and endocrine responses: A randomized, three-way crossover study in healthy volunteers. *Crit Care Med* 2010; **38**(2): 464-70.
37. Todd SR, Malinoski D, Muller PJ, Schreiber MA. Lactated Ringer's is superior to normal saline in the resuscitation of uncontrolled hemorrhagic shock. *J Trauma* 2007; **62**(3): 636-9.
38. Reid F, Lobo DN, Williams RN, Rowlands BJ, Allison SP. (Ab)normal saline and physiological Hartmann's solution: A randomized double-blind crossover study. *Clin Sci (Lond)* 2003; **104**(1): 17-24.
39. Wilkes NJ, Woolf R, Mutch M, et al. The effects of balanced versus saline-based hetastarch and crystalloid solutions on acid-base and electrolyte status and gastric mucosal perfusion in elderly surgical patients. *Anesth Analg* 2001; **93**(4): 811-6.
40. Scheingraber S, Rehm M, Sehmisch C, Finsterer U. Rapid saline infusion produces hyperchloremic acidosis in patients undergoing gynecologic surgery. *Anesthesiology* 1999; **90**(5): 1265-70.
41. McFarlane C, Lee A. A comparison of plasmalyte 148 and 0.9% saline for intra-operative fluid replacement. *Anaesthesia* 1994; **49**(9): 779-81.
